# AI for Intelligent Transportation Systems: A Systematic Review of Applications in Demand-Responsive Transport

**DOI:** 10.3390/s26154945

**Published:** 2026-08-05

**Authors:** Sarah Di Grande, Thamires de Souza Oliveira, David Pagano, Salvatore Cavalieri

**Affiliations:** Department of Electrical, Electronic and Computer Engineering, University of Catania, 95125 Catania, Italy; t.desouzaoliveira@unict.it (T.d.S.O.); david.pagano@phd.unict.it (D.P.); salvatore.cavalieri@unict.it (S.C.)

**Keywords:** demand-responsive transport, machine learning, predictive modelling, forecasting, intelligent transportation systems

## Abstract

**Highlights:**

**What are the main findings?**

- Machine Learning-based predictive analytics in Demand-Responsive Transport is emerging as a multi-level decision-support tool, supporting not only demand forecasting, but also operational reliability, service management, and planning-oriented decisions.
- The literature has expanded rapidly in recent years; however, substantial heterogeneity remains in data sources, modelling approaches, validation strategies, and implementation readiness. Indeed, most models were evaluated retrospectively, offline, or through simulations, while prospective deployment and evaluation in live DRT operations remained limited. The methodological assessment showed generally favorable predictor quality and low risk of bias for outcomes, but high risk in the analysis domain in just over half of the studies.

**What are the implications of the main findings?**

- This review provides a structured reference to help researchers, transport planners, and decision-makers comparing data sources, predictive methods, and evaluation strategies according to specific Demand-Responsive Transport applications.
- Future studies should combine transparent data, robust baselines, multiple evaluation metrics, and prospective field assessment to establish the operational effectiveness of Machine Learning-based predictive models in Demand-Responsive Transport.

**Abstract:**

Demand-Responsive Transport (DRT) has emerged as a flexible public transport strategy to improve accessibility, service coverage, and operational efficiency in contexts where conventional fixed-route services are inefficient, insufficient, or difficult to operate. In parallel, Artificial Intelligence (AI), and particularly Machine Learning (ML), has increasingly been investigated for predictive tasks relevant to DRT planning and operations, including demand forecasting, travel-time estimation, service reliability assessment, and decision support. This study presents a systematic literature review of ML-based predictive analytics in public-transport-oriented DRT services. After the screening and eligibility process, the included studies were analyzed according to predictive task, data source, modelling technique, validation strategy, performance metrics, and implementation-related challenges; methodological quality, risk of bias (RoB), and applicability were assessed using a PROBAST+AI-based framework. The results show that research in this field has expanded rapidly in recent years and is moving from isolated demand prediction models towards more integrated frameworks linking prediction, optimization, and service planning. However, the evidence remains fragmented, with substantial heterogeneity in data sources, spatial and temporal scales, modelling approaches, and evaluation procedures. The quality assessment showed generally favorable predictor quality and low outcome-related RoB, but analysis-related RoB was high in just over half of the studies, mainly because independent evaluation and validation accounting for temporal, spatial, or simulation-induced dependence were often lacking. Most studies provided retrospective, offline, simulation-based, or conceptual decision-support evidence, whereas prospective field deployment, external validation, and post-implementation monitoring were rarely or insufficiently documented. This review therefore provides a structured synthesis of current ML research in DRT and identifies priorities for future work, including improved reproducibility, stronger validation, robust baseline comparisons, multiple evaluation metrics, greater interpretability, and prospective assessment under real-world operational conditions.

## 1. Introduction

Sustainable, inclusive, and accessible mobility has become a central priority for contemporary transport systems [1,2]. Public transport authorities are increasingly required to provide services that are not only efficient and environmentally sustainable, but also capable of responding to heterogeneous mobility needs across different territories and population groups. In this scenario, Demand-Responsive Transport (DRT) has gained increasing attention as a flexible form of public transport that can complement conventional fixed-route and fixed-schedule services by adapting routes, stops, or schedules according to user demand and operational constraints [3]. DRT is particularly relevant in contexts where traditional public transport is difficult to operate efficiently, such as low-density, rural, peri-urban, or poorly connected areas, as well as in services designed for specific user groups, including paratransit users, older adults, people with disabilities, commuters, and campus communities [4]. Recent evidence indicates that DRT is expanding as an operational service model. More than 1600 DRT services were launched worldwide between 2019 and 2024, while a market forecast cited in recent research projected annual sector growth of approximately 56% through 2030 [5]. This expansion is particularly relevant to the global decarbonization agenda. Transport accounts for approximately 23% of global energy-related CO_2_ emissions, with road vehicles responsible for about 70% of direct transport emissions [6]. At the individual level, living car-free has been identified as one of the transport-related consumption changes with the greatest climate-change mitigation potential [7]. In this context, DRT may support modal shift by providing a more flexible and user-responsive alternative to private car use and conventional fixed-route public transport. Although outcomes depend on service design and operating conditions, specific DRT configurations reviewed in the literature have been associated with reductions of approximately 15–20% in vehicle travel distance and about 78% in average passenger waiting time compared with conventional services [8,9]. DRT has also expanded markedly as a research domain. A bibliometric analysis of the Web of Science Core Collection identified 468 DRT-related publications between 2000 and 2025 and found a pronounced increase in annual research output after 2020. However, the literature remained unevenly distributed across application contexts, with urban scenarios receiving considerably more attention than rural and intercity settings [10]. Previous systematic evidence also revealed a strong methodological imbalance. Of the 231 DRT studies reviewed by Schasché et al. [11], only 44 (19.0%) adopted a socio-scientific approach, indicating that economic and mathematical perspectives have predominated, while user-centered questions such as acceptance have received comparatively limited attention.

From the perspective of Intelligent Transportation Systems (ITS), DRT represents a promising but complex mobility solution. Its potential lies in the possibility of integrating flexible service design with digital platforms, real-time monitoring, and data-driven decision support. The diffusion of app-based booking systems, automated reservation platforms, vehicle location technologies, mobile devices, smart cards, Wi-Fi traces, Global Positioning System (GPS), and traffic sensors has increased the availability of heterogeneous mobility data that can be used to observe, model, and predict travel behavior [3,10]. In this sense, modern DRT systems are closely connected to sensor-based and sensor-derived data streams, which may provide information on passenger demand, vehicle trajectories, travel times, waiting times, stop locations, traffic conditions, and spatial patterns of mobility. These data sources represent a key enabler of more adaptive public transport services, as they allow operators to identify underserved areas, anticipate demand peaks, reduce inefficient vehicle movements, and improve accessibility for users who are not adequately served by conventional fixed-route networks.

At the same time, the practical implementation of DRT remains challenging. DRT operations are intrinsically dynamic and uncertain: passenger demand is stochastic and time-varying, requests may arrive in real time, users may cancel reservations or fail to show up, and service quality must be balanced against operational efficiency under strict constraints. These constraints may include vehicle capacity, pick-up and drop-off time windows, maximum waiting time, maximum ride time, detour limits, driver availability, and fleet size [12,13,14,15,16]. Moreover, the benefits of DRT are not automatic. Poorly designed services may generate high operating costs, low vehicle occupancy, long waiting times, or limited integration with the existing public transport network [11,17,18,19]. Beyond these operational aspects, successful adoption also depends on whether the service is accessible and usable by the populations it is intended to support. Digital booking interfaces may create barriers for older adults and users with limited digital access or literacy, while age, gender, and income may influence DRT adoption and the risk of transport exclusion. Therefore, the successful deployment of DRT requires not only robust planning, reliable demand estimation, and operational strategies able to adapt to spatial and temporal variability, but also attention to accessibility, user inclusion, and the distribution of service benefits [3,20]. Metaheuristic methods have gained increasing relevance in transportation addressing complex optimization, planning, scheduling, resource-allocation, and demand-management problems [21,22], which are also central to the design and operation of DRT services.

In this context, Artificial Intelligence (AI)-based approaches, particularly Machine Learning (ML) and Deep Learning (DL), have been increasingly adopted to support DRT planning, monitoring, and management, with predictive modelling emerging as a key component of data-driven decision support. Existing studies have used ML-based approaches for several predictive purposes, including demand forecasting, reservation prediction, travel-time estimation and Estimated Time of Arrival (ETA), user and trip profiling, spatiotemporal demand modelling, service reliability prediction, stop or point-of-interest (POI) classification, and decision support for routing, dispatching, vehicle rebalancing, and fleet sizing [12,14,23,24,25]. Within this spectrum, predictive modelling plays a fundamental role because accurate predictions of demand, travel time, and service reliability can inform both real-time operational decisions and longer-term planning strategies. For example, demand forecasts can support vehicle pre-positioning, capacity allocation, request insertion, and dynamic routing, while planning-oriented predictive models can help identify areas where DRT may improve accessibility, reduce service gaps, and complement existing public transport.

Previous contributions have examined topics related to, but distinct from, the scope of the present review. Schasché et al. [11] systematically reviewed DRT research with a particular focus on user acceptance, conflicting service expectations, and the specific challenges of rural areas. Deka et al. [26] provided a review-oriented perspective on the evolution of DRT towards more sustainable services, drawing on global cases, bibliometric evidence, and a case study of Bus-on-Demand in Dubai. Buics et al. [27] examined the role of DRT in sustainable urban mobility through a systematic literature review and stakeholder analysis, focusing on technological developments, implementation challenges, and stakeholder perspectives. None of these studies specifically reviewed ML-based predictive modelling in DRT. From an ML perspective, Liu et al. [28] surveyed ML applications in commercial ride-hailing services, including spatiotemporal analysis, travel behavior, order matching, and vehicle dispatching. Alexandre et al. [29] reviewed ML applications in public bus transportation across several operational problems, including travel-time and passenger-flow prediction. Di Torrepadula et al. [30] systematically reviewed ML methods for public transportation demand prediction more broadly, without focusing specifically on DRT services or on the wider range of operational and planning-oriented predictive tasks considered in the present study. Taken together, these reviews examine ML in adjacent transport domains, but they do not treat public-transport-oriented DRT as a distinct field or jointly analyze its forecasting, operational, and planning-oriented predictive applications.

Therefore, despite the growing body of research on predictive modelling for flexible and on-demand mobility, the evidence remains fragmented. In line with the policy-oriented aim of this review, we adopt a public-transport-oriented perspective on DRT. This review does not aim to cover the broader domain of on-demand or shared mobility services. Rather, it focuses on demand-responsive services that are conceived, planned, or operated as part of collective mobility provision, and that are intended to complement, reinforce, or partially substitute fixed-route and fixed-schedule public transport when conventional services are inefficient, insufficient, or difficult to operate. This includes flexible bus services, dial-a-ride (DAR), paratransit, microtransit, and other on-demand public transport schemes when they are relevant to public mobility planning, service accessibility, or local transport provision.

This boundary is not merely terminological, but it is also justified by the different institutional and operational logic underlying public-transport-oriented DRT and commercial platform-based mobility services. While public DRT is generally framed as flexible collective transport solution designed to complement, replace, or reinforce fixed-route public transport, especially where conventional services are inefficient or insufficient, ride-hailing and other platform-based services are more commonly organized around market-mediated access, individual demand–supply matching, vehicle availability, pricing, user convenience, and commercial service provision [31]. Therefore, although such services may also rely on on-demand requests, dynamic allocation, or ML-based predictive algorithms, distinguishing them from public-transport-oriented DRT is methodologically relevant, as the two service logics imply different policy objectives, operational constraints, prediction targets, performance indicators, and equity implications [32]. Accordingly, studies focusing exclusively on these services are outside the main scope of this review when they are not explicitly guided by the public transport function of the DRT.

Within the scope of this review, studies differ substantially in terms of DRT context, prediction task, data source, spatial and temporal scale, model architecture, validation strategy, and performance metrics. Some studies rely on real-world operational data, such as reservation logs, GPS trajectories, smart-card transactions, or vehicle tracking data, whereas others use synthetic demand, simulation outputs, or proxy mobility data. Similarly, model evaluation is not always performed using comparable or deployment-oriented protocols, and it is often unclear whether validation strategies adequately reflect the temporal and spatial uncertainty that characterizes real DRT operations. This fragmentation makes it difficult for researchers, transport planners, and urban decision-makers to identify which ML methods and data sources are most suitable for specific DRT-related tasks, such as demand forecasting, service planning, operational management, or performance evaluation.

A systematic review of ML applications in DRT is therefore needed for both scientific and practical reasons. From a research perspective, such a review can clarify how predictive tasks have been formulated, which algorithms have been used, how sensor-derived and operational data have been incorporated, and how model reliability has been assessed. From a policy and planning perspective, it can provide evidence to support the design, introduction, or improvement of DRT services within local public transport systems. This is especially important because DRT remains an emerging and unevenly implemented mobility solution, although it has the potential to improve the flexibility, efficiency, and inclusiveness of public transport when appropriately integrated into broader ITS frameworks.

Accordingly, this systematic review aims to provide a structured synthesis of ML-based predictive modelling applications in public-transport-oriented DRT. In this review, predictive modelling is used as an umbrella term that includes both forecasting tasks, defined as time-oriented predictions of future demand or service patterns, and other prediction-oriented applications supporting DRT planning, monitoring, and operations. The remainder of the paper is organized as follows. Section 2 describes the materials and methods, including the search strategy, eligibility criteria, study selection process, data-extraction framework, methodological quality and a quantitative synthesis of study characteristics. Section 3 presents a general analysis of the reviewed studies, and Section 4 organizes the evidence into three application domains: demand forecasting, operational predictive analytics, and planning-oriented analytics. For each domain, the review summarizes predictive tasks, data sources, input features, modelling approaches, validation strategies, and performance metrics. Relevant gaps in the literature and future directions are also highlighted. Finally, Section 5 presents the main conclusions and outlines future research directions.

The research questions (RQs) reported in Table 1 and Table 2 guide this review and provide a framework for organizing the analysis.

## 2. Materials and Methods

The protocol established for the present systematic review was registered and is available in full elsewhere [33].

A systematic literature search was carried out in Scopus, Web of Science, and IEEE Xplore to identify studies applying ML for predictive modelling in DRT systems. The final search was conducted on 15 April 2026.

The search strategy was designed around three concept blocks: DRT service contexts, forecasting/prediction tasks, and ML methods. The DRT block included synonyms and closely related service labels (e.g., demand-responsive transport/transit, dial-a-ride, paratransit, microtransit, on-demand public transit, flexible transit). The predictive modelling block targeted prediction-oriented outcomes relevant to DRT planning and management, including demand/request forecasting, ridership prediction, travel-time/ETA prediction, wait-time prediction, and cancellation/no-show prediction. The ML block captured both general and model-specific terminology (e.g., ML, AI, DL, neural networks, and representative algorithms). The prediction block was intentionally centered on forecasting- and prediction-related terminology, because the aim of the review was to identify studies in which ML was explicitly used to predict DRT-related demand, service outcomes, or planning-relevant conditions. Broader ML applications in DRT that did not include an explicit predictive component were therefore outside the scope of the search strategy.

The search strategy was developed in Scopus and then adapted to the syntax of the other databases while preserving the same conceptual structure. The Scopus string is: “TITLE-ABS-KEY ((“demand responsive” W/2 (transport OR transit OR transportation OR bus OR service)) OR “demand-responsive transit” OR “demand-responsive transport” OR “dial-a-ride” OR “dial a ride” OR paratransit OR microtransit OR “on-demand transit” OR “on demand transit” OR (“flexible” W/1 (transit OR transport OR bus))) AND TITLE-ABS-KEY (forecast* OR predict* OR “demand prediction” OR “demand forecasting” OR ridership OR “request prediction” OR “trip demand” OR “travel time prediction” OR “arrival time” OR ETA OR “estimated time of arrival” OR “wait time” OR “waiting time” OR “no-show” OR noshow OR cancellation*) AND TITLE-ABS-KEY (“machine learning” OR “deep learning” OR “artificial intelligence” OR “neural network*” OR LSTM OR GRU OR CNN OR transformer* OR “random forest” OR “gradient boosting” OR XGBoost OR LightGBM OR “support vector” OR SVM OR “gaussian process” OR bayesian)”.

The Preferred Reporting Items for Systematic Reviews and Meta-Analyses (PRISMA) guidelines were followed for study identification, screening, eligibility assessment, and inclusion [34]. The completed PRISMA 2020 checklist is provided in Supplementary Table S1. Eligibility criteria were defined a priori to identify studies addressing predictive ML applications in public-transport-oriented DRT or closely related services explicitly framed as DRT, paratransit, DAR, microtransit, on-demand transit, or flexible transit. Eligible studies had to apply ML methods for forecasting or predictive analytics and had to address at least one DRT-related predictive task, such as demand forecasting, travel-time prediction, waiting-time prediction, reservation-outcome prediction, service-quality prediction, or planning-oriented suitability assessment.

Studies were excluded if they were unrelated to DRT or conceptually equivalent collective mobility services, if they focused solely on routing, scheduling, optimization, or simulation without a predictive ML component, or if they addressed commercial taxi, ride-hailing, car-sharing, or shared-mobility services without an explicit DRT-related public transport objective. Studies using taxi, smart-card, Wi-Fi, shared-mobility, or other proxy mobility datasets were included only when these data were explicitly used to model, simulate, or support DRT, microtransit, flexible transit, or on-demand public transport scenarios. Review articles, editorials, abstract-only publications, non-peer-reviewed records, studies without sufficient methodological information, studies for which the full text could not be retrieved, and non-English publications were also excluded. Eligible records were limited to peer-reviewed journal articles and conference papers.

All retrieved records were managed and screened using Rayyan (https://www.rayyan.ai/; accessed on 15 April 2026), a web-based tool supporting systematic review screening and deduplication [35]. After duplicate removal, two researchers screened the records. Disagreements were resolved by consulting a third researcher to make the final decision. The first screening was conducted in two sequential stages: title screening and abstract screening of the retained records. For all studies passing the abstract stage, the full text was retrieved and assessed against the eligibility criteria. This process resulted in the final list of included studies.

The full texts of all included articles were reviewed to extract the information needed to answer the predefined RQs. For each included study, we extracted information on the first author, year of publication, study title, publication type, conference or journal name, type of DRT service, predictive task, geographical location, data sources, temporal coverage of the data used for modelling, model adopted, comparison model(s), validation strategy, performance metrics, important variables, key findings, operational context, and ultimate objectives.

The methodological quality, applicability, and Risk of Bias (RoB) of the included studies were assessed at the domain level using an adapted PROBAST+AI framework [36]. The assessment distinguished between prediction model development and model evaluation and considered four domains: data sources, predictors, outcome, and analysis. For model development, data sources, predictors, and outcomes were assessed for quality and applicability, while the analysis domain was assessed for quality only. For model evaluation, the same three domains were assessed for RoB and applicability, while analysis was assessed for RoB only. Data-source quality considered reliability, representativeness, sample size, completeness, and spatial and temporal coverage. Predictor quality considered whether variables were clearly defined, consistently determined, available at the intended prediction time, and processed without using future or evaluation-set information. Outcome quality considered the clarity, consistency, objectivity, and appropriateness of the predicted target. The analysis domain considered preprocessing, missing-data handling, sample size, model specification, hyperparameter tuning, class imbalance, comparator models, validation design, separation between model selection and final testing, and the appropriateness of performance measures. For spatially or temporally structured data, the assessment also considered whether dependence between observations was appropriately addressed. Applicability was judged relative to AI-based predictive analytics for public-transport-oriented DRT, considering the correspondence of the data and service context, the practical availability of the predictors, and the relevance of the outcome to DRT demand, operations, service quality, or planning. Each domain was rated as Low, High, or Unclear concern. Low indicated no major concern, High indicated a limitation likely to affect validity, applicability, or performance estimates, and Unclear indicated insufficient reporting. The main modelling pipeline was used as the unit of assessment; when several algorithms shared the same data and analytical procedure, the judgement referred to the overall process leading to the selected model. Each judgement was supported by a study-specific justification based on the full text of the relevant included study, data-extraction table, and any available supplementary material or correction associated with that study. Complete judgements and percentage distributions are reported in the corresponding tables and figures.

A synthesis was then performed in two stages. First, a general descriptive analysis was conducted to provide an overview of the included studies, considering publication year, publication type and venue, geographical distribution of the case studies, temporal coverage, type of data used for modelling, and ML approaches. To complement the descriptive analysis, we conducted a quantitative synthesis of the 23 included studies by application group, main model family, validation strategy, data availability, temporal coverage, and data provenance. Each study was assigned to a single primary application group and a single main model family based on the principal function of its predictive output and the model identified by the authors as the main approach. To avoid double counting, validation strategies were classified into mutually exclusive categories based on the reported data-partitioning and resampling procedures, with temporal or spatial independence taking precedence when explicitly implemented. Data availability was classified according to whether the core modelling data were publicly accessible, restricted or non-public, or derived from a combination of public and non-public sources. Data provenance was classified as either direct operational data obtained from DRT or closely related public transport services or proxy, indirect, or simulation-based data. Two cross-tabulation analyses were subsequently conducted. The first examined the relationship between data availability and data provenance. Percentages were calculated within each data-availability category, whereas the “Total” row reports the overall distribution across all 23 studies. The second examined the relationship between temporal coverage and data provenance. Temporal coverage was classified as short-term (≤2 months), medium-term (>2 to 12 months), long-term (>12 months), or unclear/not applicable. For this cross-tabulation, percentages were calculated within each data-provenance category, allowing the distribution of observation periods to be compared between studies using direct operational or closely related public transport data and those relying on proxy, indirect, or simulation-based data.

Second, a thematic analysis was carried out by grouping the studies according to their main predictive application. When studies could fit more than one category, they were assigned according to the primary function of the predictive output within the study. Three groups were identified: forecasting, operational predictive analytics, and planning-oriented predictive analytics. This three-part classification was used to distinguish studies according to whether ML-based prediction was mainly applied to anticipate future service conditions over time, support day-to-day operational decisions, or inform strategic and tactical planning. Therefore, forecasting studies included papers in which ML models were used for temporally oriented prediction, namely to anticipate future demand, reservations, origin–destination (OD) movements, request volumes, or other service conditions over a defined forecasting horizon. Operational predictive analytics included studies using ML-based prediction to support service management, monitoring, routing, request handling, ETA, waiting-time prediction, or other operational decisions. Planning-oriented predictive analytics included studies in which predictive models supported strategic or tactical decisions, such as identifying suitable areas for DRT deployment, estimating service capacity, modelling spatial demand patterns, or informing service design.

The studies within each group were then analyzed to compare their data sources, modelling strategies, validation procedures, evaluation metrics, key findings, operational objectives and research gaps. This approach was intended to support a task-oriented interpretation of the literature and to help researchers, practitioners, and urban decision-makers identify the most appropriate techniques, data sources, and methodological choices that are most relevant to the specific objective they aim to address.

## 3. Analysis of the Reviewed Studies

**Figure 1:**
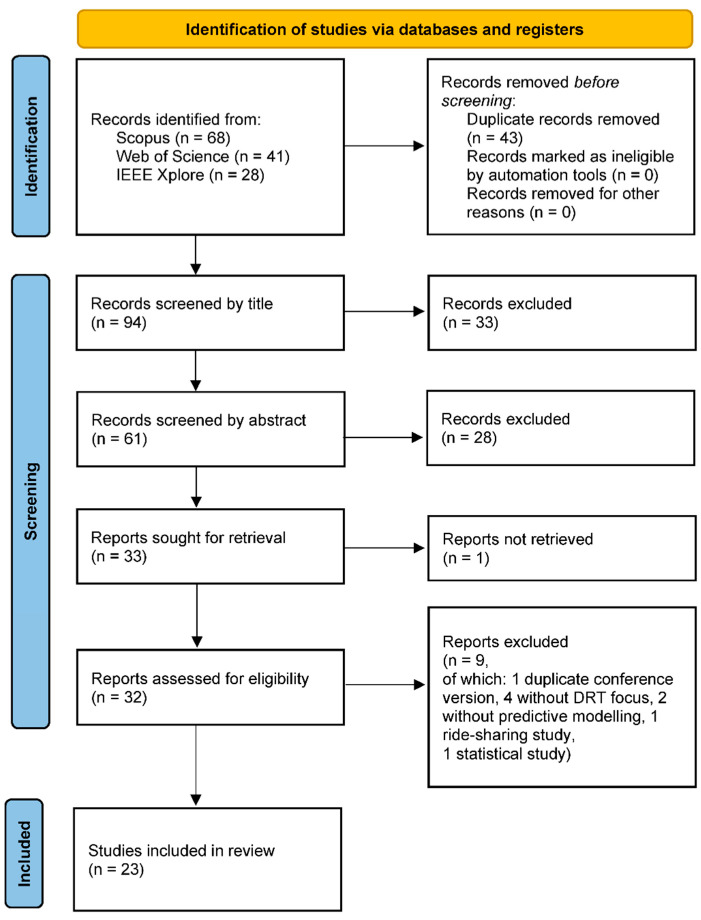
PRISMA flow diagram summarizing the literature search and study selection process, from database identification and duplicate removal to title/abstract screening, full-text eligibility assessment, and final inclusion in the systematic review.

The study selection workflow is presented in Figure 1.

The database search identified a total of 137 records: 68 from Scopus, 41 from Web of Science, and 28 from IEEE Xplore. After duplicate removal, 94 records remained. Title screening led to the exclusion of 33 records because they were related to transport but not to DRT (*n* = 8), were conference reviews (*n* = 11), literature reviews (*n* = 1), or concerned unrelated domains, including energy (*n* = 4), public health (*n* = 2), supply chain (*n* = 2), telecommunications (*n* = 1), port operations (*n* = 1), robotics (*n* = 1), aviation (*n* = 1), and semiconductors (*n* = 1); 61 records were retained for the next screening. After abstract screening, 28 further records were excluded because they were not related to DRT (*n* = 8), did not include predictive modelling (*n* = 12), or focused on unrelated topics, including energy (*n* = 2), ride-hailing (*n* = 2), telecommunications (*n* = 1), sustainability (*n* = 1), unmanned aerial vehicles (*n* = 1), or were service-evaluation studies based on statistical analysis without an ML-based predictive analytics component (*n* = 1). As a result, 33 articles were eligible for full-text assessment.

During full-text assessment, 10 articles were excluded. One article was excluded because the full text could not be retrieved, and another because it was the conference version of an already included extended journal article, in which the methodological details had already been fully reported, while the remaining exclusions were due to lack of relevance to the review scope: no DRT focus (*n* = 4), no predictive modelling component (*n* = 2), ride-sharing focus (*n* = 1), and statistical analysis without an ML-based predictive component (*n* = 1). This resulted in a final sample of 23 included studies [12,13,14,15,16,23,24,25,37,38,39,40,41,42,43,44,45,46,47,48,49,50,51].

**Figure 2:**
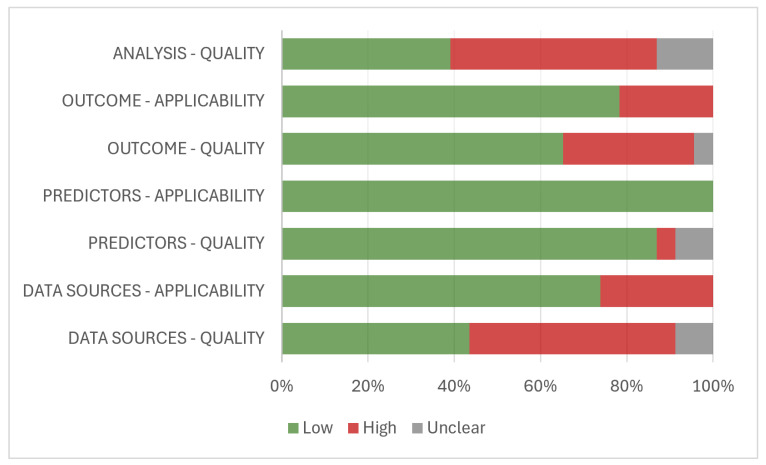
Percentage distribution of quality judgments for prediction model development. Green, red, and grey segments indicate Low, High, and Unclear concerns, respectively.

**Figure 3:**
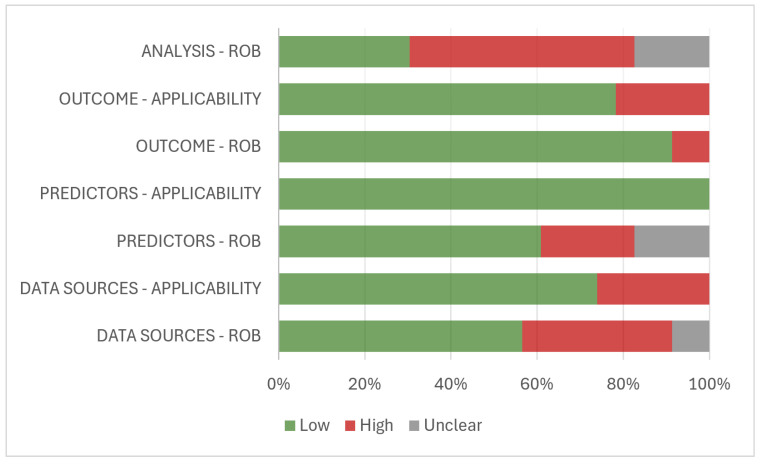
Percentage distribution of RoB judgments for prediction model evaluation. Green, red, and grey segments indicate Low, High, and Unclear concerns, respectively.

The domain-level assessment revealed substantial variation in the methodological quality and RoB of the 23 included studies. The complete judgements are reported in Table 3 and Table 4, while Figure 2 and Figure 3 present their percentage distributions.

Regarding prediction model development, data-source quality was rated as Low in 10 studies (43.5%), High in 11 (47.8%), and Unclear in 2 (8.7%). High concerns reflected different limitations. Peled et al. [23] used a short series of Wi-Fi-derived movements as a proxy for shuttle demand, while Liyanage et al., Khair et al., Tandan et al., Veve et al., and Oh et al. [38,39,41,46,47] relied wholly or partly on fixed-route, taxi, or simulated data rather than direct observations from the intended DRT service. Wu et al. and Li et al. [15,44] used only approximately one month of operational data, whereas Ukam et al. examined a relatively small sample. Shen et al. [45] combined one month of paratransit demand with census information from a different temporal reference, and Marković et al. [50] primarily used simulated planning scenarios. Data-source quality was Unclear for Portell et al. and Kim et al. [16,48] because the exact temporal coverage or temporal alignment of the modelling data was not sufficiently reported.

Predictor quality was generally favorable, being rated as Low in 20 studies (87.0%), High in 1 study (4.3%), and Unclear in 2 studies (8.7%). The High judgement for Putri et al. [40] reflected limited reproducibility of the clustering, normalization, and observation-selection procedures and insufficient information on whether preprocessing was separated from testing. Predictor quality was Unclear for Liyanage et al. [38] because standardization was described before data partitioning, and therefore it is not clear if it was calculated on the entire dataset, and for Park et al. [42] because the temporal alignment of operational and spatial indicators with the prediction time was not fully documented.

Outcome quality was rated as Low in 15 studies (65.2%), High in 7 (30.4%), and Unclear in 1 (4.3%). In Peled et al., Khair et al., and Veve et al. [23,39,46], the target was based on indirect mobility or demand proxies. Putri et al. [40] used manually assigned stop-type labels without reporting inter-rater agreement or independent verification, while Tandan et al. [41] used assign/reject labels generated by a simulated dispatching process. Kim et al. [48] defined service-suitability classes through operator-informed usage thresholds that were not externally validated. In Marković et al. [50], the capacity outcomes were generated by a routing heuristic rather than directly observed or proven-optimal resource requirements. Outcome quality was Unclear for Imhof et al. [51] because cumulative pick-up and drop-off counts were compared across services with incompletely reported and potentially different observation periods.

Analysis quality was the domain with the largest number of methodological concerns: 9 studies (39.1%) were rated Low, 11 (47.8%) High, and 3 (13.0%) Unclear. Zigrand et al. and Wu et al. [14,15] did not clearly report a validation set dedicated to model selection, while Khair et al. [39] provided limited information on tuning, baselines, and the independence of the evaluation procedure. Putri et al., Ukam et al., Tandan et al., Park et al., and Oh et al. [25,40,41,42,47] relied on internal or randomly partitioned evaluations that did not adequately account for repeated temporal observations, common routes, neighboring spatial units, or simulation dependence. Belhaiza et al. [43] compared multiple neural-network configurations using the same evaluation data, Shen et al. [45] performed variable selection and model estimation without an independent final test, and Veve et al. [46] developed and assessed the clustering and line-design procedure on the same selected sample. Analysis quality was Unclear for Liyanage et al., Kim et al., and Imhof et al. [38,48,51] because the relationships among preprocessing, model selection, cross-validation, and final evaluation were not sufficiently described.

Applicability was more favorable than methodological quality. Predictor applicability was rated as Low concern in all 23 studies, indicating that the investigated operational, temporal, spatial, socioeconomic, and built-environment variables were generally considered obtainable in the intended planning or operational settings. High concern for data-source applicability was assigned to Peled et al., Liyanage et al., Khair et al., Tandan et al., Veve et al., and Oh et al. [23,38,39,41,46,47], mainly because their data represented campus movements, fixed-route ridership, taxi trips, shared mobility, or simulated demand rather than the intended operational DRT context. Outcome applicability was also rated High for Peled et al., Liyanage et al., Khair et al., Veve et al., and Oh et al. [23,38,39,46,47], as their predicted outcomes did not directly represent observed demand or performance in the DRT service of interest.

For model evaluation, data-source RoB was rated as Low in 13 studies (56.5%), High in 8 (34.8%), and Unclear in 2 (8.7%). High judgements were assigned to Liyanage et al., Khair et al., Putri et al., Ukam et al., Tandan et al., and Oh et al. [25,38,39,40,41,47] because their evaluation samples were derived from the same internally partitioned, proxy, simulated, route-specific, or spatially correlated datasets used for development. Shen et al. and Veve et al. [45,46] did not use an independent holdout sample. RoB was Unclear for Belhaiza et al. [43] because the test-assignment procedure was insufficiently reported and for Kim et al. [48] because the relationship between the declared train-test split and the 10-fold cross-validation was not fully explained.

Predictor-related RoB was Low in 14 studies (60.9%), High in 5 (21.7%), and Unclear in 4 (17.4%). High concerns in Khair et al., Putri et al., Ukam et al., Tandan et al., and Oh et al. [25,39,40,41,47] related to possible use of evaluation-set information during preprocessing, dependence between predictors and simulation-generated outcomes, or strong similarity between training and test observations. The judgement was Unclear for Zigrand et al., Liyanage et al., Park et al., and Shen et al. [14,38,42,45], as the available reporting did not establish whether aggregation, normalization, index construction, or variable selection had been performed exclusively within the development data.

Outcome-related RoB was comparatively limited, with 21 studies (91.3%) rated Low and 2 (8.7%) rated High. Putri et al. [40] received a High judgement because the manually assigned reference labels were subjective and no independent or blinded verification was reported. Veve et al. [46] was rated High because recurrent demand and cluster quality were determined from the same data and algorithm used to construct the proposed microtransit solution, without assessment against future or independent demand. In the remaining studies, the outcome was generally determined using the same objective or prespecified rule in development and evaluation and was not influenced by the model predictions.

Analysis-related RoB was rated as Low in 7 studies (30.4%), High in 12 (52.2%), and Unclear in 4 (17.4%). High concerns were identified for Zigrand et al., Wu et al., Liyanage et al., Khair et al., Putri et al., Ukam et al., Tandan et al., Park et al., Belhaiza et al., Shen et al., Veve et al., and Oh et al. [14,15,25,38,39,40,41,42,43,45,46,47]. The recurring reasons were lack of a clearly independent final evaluation, use of test performance during model or architecture selection, preprocessing conducted before data partitioning, and validation designs that did not adequately reflect temporal, spatial, route-level, or simulation-related dependence. The judgement was Unclear for Li et al., Kim et al., Alsaleh et al., and Imhof et al. [44,48,49,51], because it could not be established whether clustering, target construction, standardization, or model selection had been confined to the training data or appropriately separated from final testing.

Applicability concerns during model evaluation followed a pattern similar to that observed for model development. Evaluation data-source applicability was rated as Low concern in 17 studies (73.9%) and High concern in 6 studies (26.1%). High concerns were assigned to Peled et al., Liyanage et al., Khair et al., Tandan et al., Veve et al., and Oh et al. [23,38,39,41,46,47], because the evaluation samples were based on campus Wi-Fi movements, fixed-route ridership, taxi data, shared-mobility demand, or simulated scenarios rather than observations from the intended operational DRT context. Predictor applicability was rated as Low concern in all 23 studies, indicating that the evaluated temporal, operational, spatial, socioeconomic, and built-environment variables were generally considered available in the intended planning or operational setting. Outcome applicability was rated as Low concern in 18 studies (78.3%) and High concern in 5 studies (21.7%). High concerns were identified for Peled et al., Liyanage et al., Khair et al., Veve et al., and Oh et al. [23,38,39,46,47], because the evaluated outcomes represented proxy mobility, fixed-route demand, taxi demand, or algorithmically derived demand patterns rather than directly observed DRT demand or operational performance.

### 3.1. Years of Publication

**Figure 4:**
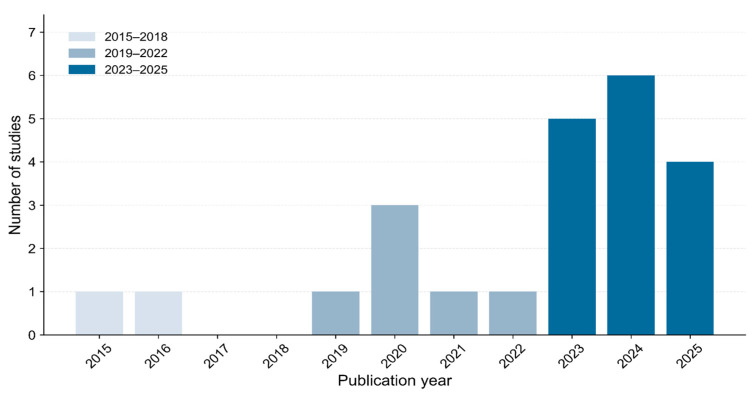
Distribution of included studies by year of publication. The color gradient indicates three publication periods, highlighting the increasing concentration of studies in the most recent years.

Figure 4 describes the distribution of publications by year. The included studies were published between 2015 and 2025, indicating that the application of ML and predictive analytics to DRT is a relatively recent and emerging research area. Only a limited number of studies were published before 2020, with one study in 2015 [45], one in 2016 [50], and one in 2019 [23]. From 2020 onwards, the number of publications increased substantially, accounting for 20 of the 23 included studies.

A particularly marked growth was observed in the most recent period. Indeed, studies published between 2023 and 2025 represented approximately two thirds of the included literature, with the highest number of publications observed in 2024. This temporal trend suggests a growing interest in data-driven and ML-based approaches for forecasting demand, supporting operational decision-making, and informing the planning of DRT and related flexible transport services.

### 3.2. Type and Venue of Publication

Regarding publication type and venues, the included studies were predominantly published as journal articles. Specifically, 18 out of 23 studies were published in journals [12,13,15,16,24,25,37,38,39,41,42,43,44,46,47,49,50,51], whereas five studies were published as conference papers [14,23,40,45,48]. This indicates that, within the literature on ML and predictive analytics for DRT, research outputs are mainly disseminated through journals rather than conference proceedings.

With respect to the distribution of publication venues, it was highly heterogeneous. Overall, the 23 included studies were distributed across 19 different venues, suggesting that this research area is not yet concentrated around a small number of specialized journals. The most recurrent venues were Transportation Research Part A in three included studies, Transportation Research Record in two studies, and the IEEE Intelligent Transportation Systems Conference, also with two studies. All other venues appeared only once.

Most journal articles were published in transportation-oriented journals, including different sections of the Transportation Research family, Transportation Research Record, Research in Transportation Economics, and the Journal of Advanced Transportation. However, several studies were also published in broader engineering, AI, and interdisciplinary journals, such as Expert Systems with Applications, Applied Sciences, Smart Cities, Heliyon, Cogent Engineering, IEEE Transactions on Computational Social Systems, and Arabian Journal for Science and Engineering. This variety reflects the interdisciplinary nature of the topic, which combines transport planning, service operations, predictive modelling, AI, and ITS.

### 3.3. Geographical Areas

**Figure 5:**
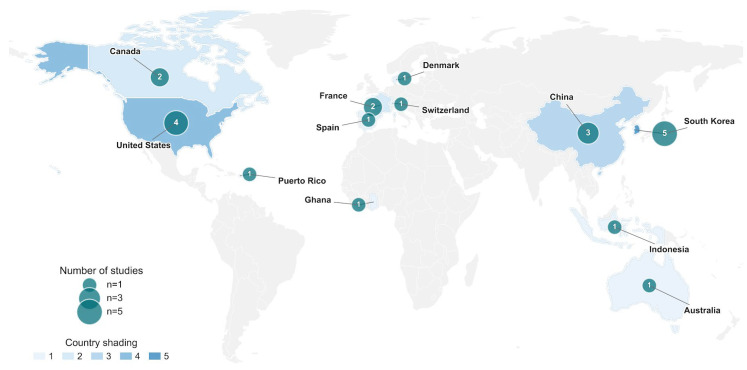
Geographical distribution of the data sources used in the included studies. Countries and territories are shaded according to the number of included studies, while bubble size also represents the study count. The number inside each bubble indicates the exact number of studies for each country or territory.

The included studies covered a broad but uneven geographical distribution in terms of data-source geography (see Figure 5). Figure 5 represents the geographical origin of the primary empirical datasets used in the included studies. Proxy mobility datasets were geographically assigned according to their actual data origin.

The largest proportion of studies was conducted in Asia [15,24,37,40,41,42,44,47,48], which accounted for 9 out of 23 studies. Within this group, South Korea was the most represented country, followed by China and Indonesia. North America and the Caribbean accounted for seven studies [13,39,43,45,46,49,50], including studies conducted in the United States, Canada, and Puerto Rico. European studies represented five of the included records [12,14,16,23,51] and were conducted in France, Denmark, Spain, and Switzerland. Only one study was conducted in Africa [25], specifically in Ghana, and one in Oceania [38], specifically in Australia.

Overall, this distribution suggests that research on ML-based prediction for DRT and related flexible transport services is geographically concentrated in Asia, North America, and Europe. In contrast, datasets and case studies from Africa, Oceania, South America, and other regions remain limited. This geographical imbalance should be considered when interpreting the generalizability of current findings, as DRT systems are strongly influenced by local mobility patterns, service regulations, urban form, public transport supply, and user needs. Furthermore, these counts include both operational DRT datasets and proxy mobility datasets and therefore should not be interpreted as representing the geographical distribution of operational DRT services. More specifically, the studies relying on proxy, indirect, or simulation-generated data were geographically associated with Spain [16], Denmark [23], Australia [38], USA [39,46,50], and South Korea [41,47].

### 3.4. Temporal Coverage of Modelling

**Figure 6:**
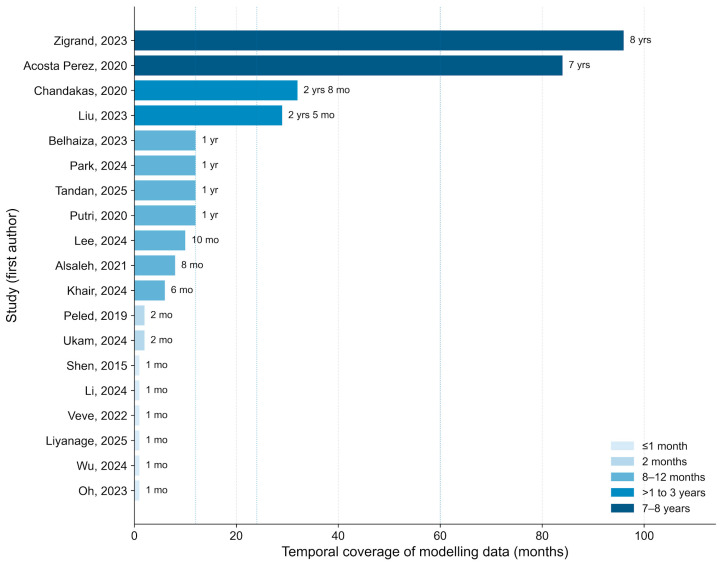
Temporal coverage of the datasets used for modelling. Each study is identified by first author and publication year, corresponding to references [12,13,14,15,23,24,25,37,38,39,40,41,42,43,44,45,46,47,49]. Bars represent studies with clearly reported dataset duration and are colored according to temporal coverage category.

The temporal coverage of the datasets used for model development varied substantially across the included studies (see Figure 6). Rather than being uniformly based on long observation periods, several studies relied on relatively short data windows. Six studies used datasets covering one month [15,38,44,45,46,47], while two additional studies used two months of data [23,25].

Medium-term datasets were also common. Seven studies used data covering approximately 6 to 12 months [37,39,40,41,42,43,49]. A smaller number of studies relied on multi-year datasets. Two studies used datasets covering more than one year and up to three years [12,24], while two studies used longer longitudinal datasets of seven or eight years [13,14].

In four studies [16,48,50,51], this information was not explicitly reported; therefore, these studies were excluded from the analysis and from Figure 6.

Overall, the temporal coverage of modelling data was heterogeneous and not always clearly reported. This is an important methodological aspect, as short observation windows may limit the ability of predictive models to capture seasonal patterns, long-term demand variability, holidays, exceptional events, and changes in service use over time. Conversely, longer datasets may provide a stronger basis for modelling recurrent demand patterns and temporal variability, although they may also include changes in service design, user behavior, or operational conditions over time.

For this reason, temporal coverage was analyzed primarily in terms of dataset duration rather than only calendar year.

### 3.5. Data Used for Modelling

Different types of data sources have been used in the included studies. These include operational data about the DRT systems, data gathered using sensors, and spatial or contextual data. Most studies relied on actual operational data which was gathered using information like trip records, reservations, GPS tracking, waiting times, and previous demand data, which have largely been used in the modelling of demand, monitoring of performance, and support of operations [12,13,14,15,24,25,37,40,42,43,44,45,48,49,51].

A few studies supplemented their data with contextual data such as socioeconomic data, land use, accessibility, public transport supply, and weather conditions, indicating the importance of such factors in demand and performance in DRT systems.

When actual operational data was not available, proxy datasets such as taxi data, e-ticketing data, and data from shared mobility services were used to model demand or simulate DRT system operations [16,23,38,39,41,46,47,50].

### 3.6. Machine Learning Approaches

**Figure 7:**
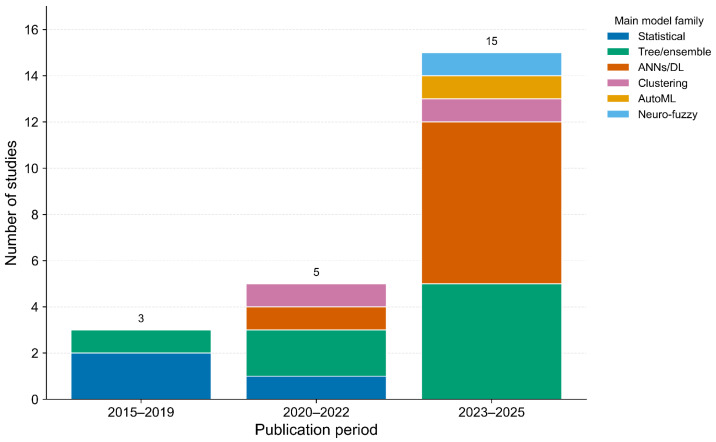
Distribution of included studies by publication period and main model family. The stacked bars show the number of studies published in each period, stratified by the main model family adopted.

The methodological differences among the papers that have been analyzed include use of statistical modelling, ML, DL, clustering methods, Automated ML (AutoML) platforms, and neuro-fuzzy algorithms (see Figure 7). Statistical techniques covered regression and time series [12,45,50], while tree-based and ensemble modelling methods were associated with Random Forest (RF), Gradient Boosting (GB), eXtreme Gradient Boosting (XGBoost), and Light Gradient Boosting Machine (LightGBM), used for working with complex non-linear data [13,16,23,24,41,48,49,51].

Neural network/DL techniques included Artificial Neural Networks (ANNs), Multilayer Perceptron (MLP), Long Short-Term Memory (LSTM) and other types of neural network-based solutions [14,15,25,37,38,39,40,43]. Clustering techniques were used less often, mostly in spatiotemporal applications [44,46]. AutoML solutions and neuro-fuzzy systems were less employed [42,47], and their usage can be seen as an indication of an increasing interest towards automatic and hybrid modelling approaches. In addition, apart from primary models, there were additional algorithms used as comparative techniques. The same techniques were used as both main and comparative models depending on the study.

Figure 7 clearly illustrates the fact that there is a definite evolution in terms of the methods applied by the literature published over time. Publications from 2015 until 2019 mostly centered on statistical and regression models. During 2020–2022, tree-based models gained dominance alongside the emergence of ANN/DL and Clustering-based models. During 2023–2025, neural-network and deep-learning models were the most frequently represented methodological family, accounting for 7 of the 15 studies published in this period (46.7%), followed by tree-based and ensemble approaches in five studies (33.3%). It is also worth noting that the emergence of AutoML techniques and neuro-fuzzy models indicates a growing level of complexity in methodology. Despite this development, ensemble models continue to have relevance due to interpretability and computation efficiency, as well as the fact that they can be used as a benchmark measure for performance.

### 3.7. Quantitative Synthesis of Study Characteristics

To complement the descriptive analysis, Table 5 provides a quantitative synthesis of the 23 included studies according to application group, main model family, validation strategy, data availability, temporal coverage, and data provenance.

The quantitative synthesis shows a relatively balanced distribution across application groups, with operational predictive analytics and planning-oriented predictive analytics each accounting for eight studies (34.8%), compared with seven demand-forecasting studies (30.4%). Considering first the overall totals reported in Table 5, neural networks and deep learning and tree-based and ensemble models were the joint most frequent model families, with eight studies each (34.8%), together representing 69.6% of the evidence base. Among the 15 studies published from 2023 onwards, neural-network and deep-learning approaches were the most frequent family, being used in seven studies (46.7%), followed by tree-based and ensemble models in five (33.3%); therefore, neural approaches represented the largest recent model family, although not an absolute majority. Validation practices remained heterogeneous. Only seven studies (30.4%) adopted time-aware evaluation, whereas 14 (60.9%) relied on conventional train–test, train–validation–test, cross-validation, or combined holdout and cross-validation procedures. Spatial transfer was assessed in only one study (4.3%), while another study did not report an independent out-of-sample evaluation. This distribution is consistent with the methodological concerns identified in the RoB assessment, particularly regarding the limited consideration of temporal, spatial, and route-level dependence. Reproducibility was also constrained by data availability, as the core modelling data were non-public, restricted, or not explicitly shared in 18 studies (78.3%). Moreover, 15 studies (65.2%) used datasets covering no more than 12 months, while only four (17.4%) used more than one year of data. Although direct operational or closely related service data were used in 15 studies (65.2%), the remaining eight (34.8%) relied on proxy, indirect, or simulation-based evidence. Overall, these results indicate that the growing methodological diversity of the field is accompanied by persistent limitations in data accessibility, temporal representativeness, and externally robust validation.

The cross-tabulation revealed clear differences among the three application groups. Demand-forecasting studies predominantly adopted neural networks and deep learning, which were used in 5 of the 7 studies (71.4%). This group also showed the greatest use of temporally appropriate evaluation, with five studies (71.4%) adopting time-aware train–test or train–validation–test procedures. Nevertheless, six forecasting studies (85.7%) relied on non-public or restricted data, and three (42.9%) used proxy, indirect, or simulation-based data. Operational predictive analytics showed greater methodological diversity: neural and tree-based models were equally represented, with three studies each (37.5%), while clustering and neuro-fuzzy methods were used in one study each. Conventional internal validation predominated in this group, accounting for 6 of the 8 studies (75.0%), although two studies adopted time-aware evaluation. Operational studies had the highest use of direct service data, with six studies (75.0%), but also the highest proportion of non-public or restricted datasets, with seven studies (87.5%). Planning-oriented predictive analytics relied mainly on tree-based and ensemble models, used in four studies (50.0%), followed by statistical models in two (25.0%). Six planning studies (75.0%) used cross-validation either alone or in combination with a holdout split; however, only one study assessed cross-area transferability, and one did not use an independent out-of-sample evaluation. Planning studies also showed the greatest uncertainty in temporal coverage, which was unclear or not applicable in three cases (37.5%). These findings confirm that model selection and evaluation design were strongly related to the intended application, while limited data accessibility, short observation periods, and predominantly internal validation remained common across the literature.

To examine whether the use of direct operational data was accompanied by greater data accessibility, Table 6 cross-tabulates data availability and data provenance. Percentages were calculated within each data-availability category, while the Total row reports the distribution across all 23 studies.

The cross-tabulation revealed a clear separation between data accessibility and operational relevance. Both studies with fully public core modelling data relied on proxy, indirect, or simulation-based data; therefore, none of the studies based on fully public datasets used direct operational DRT or closely related service data. Conversely, all three studies combining public and restricted sources used direct or closely related operational data, generally integrating non-public service records with publicly available contextual or spatial information. Among the 18 studies whose core data were non-public, restricted, or not explicitly shared, 12 (66.7%) used direct operational or closely related service data and six (33.3%) used proxy, indirect, or simulation-based evidence. Overall, although direct operational data represented the majority of the evidence base, being used in 15 studies (65.2%), such data were never fully publicly accessible. This pattern indicates a relevant trade-off between real-world operational relevance and reproducibility: studies based on direct service data may offer greater practical validity for DRT applications, but restricted access limits independent replication, external benchmarking, and comparison across services. These findings support the need for anonymized operational datasets, common data-sharing protocols, and publicly accessible benchmark datasets that better represent real DRT operations.

To examine whether temporal representativeness differed according to the type of data used, Table 7 cross-tabulates temporal coverage and data provenance.

The cross-tabulation of temporal coverage and data provenance revealed a marked concentration of proxy, indirect, or simulation-based studies within short observation periods. Five of the eight studies in this group (62.5%) used data covering no more than two months, while only two (25.0%) covered between two and twelve months; none used more than one year of data. Studies based on direct operational or closely related service data showed broader temporal coverage: 3 of 15 (20.0%) were short-term, five (33.3%) covered between two and twelve months, and four (26.7%) used more than one year of data. Accordingly, all four long-term studies in the review were based on direct operational data. Temporal coverage was unclear or not applicable in three direct-data studies (20.0%) and one proxy-data study (12.5%). Although these descriptive findings do not establish an association beyond the reviewed sample, they indicate that proxy-based evidence was particularly dependent on short observation periods, whereas direct operational data supported comparatively broader temporal coverage. Nevertheless, only 4 of the 23 studies (17.4%) provided long-term evidence, confirming that limited temporal representativeness remains a general weakness of the literature, irrespective of data provenance.

## 4. Analysis of DRT Applications

This section analyzes the three groups of papers classified according to their main predictive application. As previously outlined, these groups include forecasting, operational predictive analytics, and planning-oriented predictive analytics. By adopting this classification, the review compares studies with similar objectives to identify the most commonly used and best-performing data sources, modelling strategies, validation procedures, and evaluation metrics. Relevant research gaps are also highlighted.

### 4.1. Demand Forecasting Studies

This section summarizes the studies focused on demand forecasting. In this review, demand forecasting refers to studies in which ML models were used to anticipate future demand, reservations, OD movements, passenger volumes, or related service conditions over a defined temporal horizon.

Among the recent literature on DRT, 7 of the 23 papers focused on leveraging ML to support or improve the forecasting of demand related to DRT services. This finding is not unexpected, as the name itself, DRT, indicates that the ability to respond to changing or dynamic demand represents a key component of these services. Consequently, the accurate forecasting of demand becomes a critical driver for service optimization, enabling a more appropriate allocation of service resources, with the aim of reducing costs and/or improving service quality.

Table 8 and Table 9 summarize the key information used to analyze this group of studies.

#### 4.1.1. Subject or Use of the Prediction

The forecasting-focused papers can be further divided into three subgroups according to the intended use of the demand forecasting information. The first subgroup, comprising two papers, can be categorized as advanced operational planning and capacity management. This subgroup includes studies in which forecasting models were developed and their outputs were subsequently used for operational purposes, such as fleet sizing or driver scheduling [12,14].

The second subgroup, comprising four papers, can be categorized as adaptive routing, dispatching, and supply optimization. These studies used the outputs of forecasting models to support dynamic or adaptive decision-making processes, such as dynamic routing [15,23,37,38].

The final subgroup includes only one paper and can be classified under platform and cloud-resource management. In this case, demand forecasting data were used to determine the amount of cloud resources required to support the DRT service at any given time [39]. This study illustrates a potential extension of DRT demand forecasting to digital infrastructure management. Because this application was identified in only one study, it should be interpreted as a preliminary and emerging research direction rather than as an established trend in the DRT literature.

#### 4.1.2. Data

The next aspect to consider in the forecasting-focused papers concerns the core datasets and temporal horizons used to support demand prediction. This section examines these dimensions from an overall perspective and then discusses them in relation to the three subgroups identified above.

Across the seven forecasting papers, only two of the core demand datasets used publicly available data. The first public dataset was used by the authors of ref. [39], who employed the 2016 New York City (NYC) Yellow Cab trip record data, made available as a Kaggle dataset through Big Query on the Google Cloud Platform. This dataset is considered one of the few widely used open-source, real-world, large-scale datasets for human mobility research [52]. In ref. [15], both a numerical test and a case study were performed. The numerical test used the well-known Sioux Falls network and associated data. The Sioux Falls network was first introduced to the research community in ref. [53] as a test case for a traffic equilibrium network and has since become a de facto benchmark for evaluating algorithms in transportation science [10]. The case study, which provided a real-world application for demand forecasting, used a non-public dataset from a shared bus project conducted at the Guangzhou Higher Education Mega Center. The remaining five papers also relied on non-publicly available datasets: two were obtained from existing DRT service providers’ operational systems, two from existing public transportation operational systems, and one from an on-campus IT system providing Wi-Fi access point device probe information.

The two papers focused on advanced operational planning and capacity management adopted a daily temporal horizon for medium-term demand prediction. Study [12] used historical reservation data from an existing paratransit service provider to predict the number of completed daily reservations 7 days ahead. This forecasting horizon was selected to support vehicle planning and driver scheduling, as the labor contract required drivers to be scheduled 7 days in advance. Study [14] used data from a public transportation system containing historical travel requests with “Origin-Destination-Timeslot” information to estimate demand for the next operational day. This next-day aggregated demand was then incorporated into an offline look-ahead optimization process, which formulated driver routes on the basis of existing reservations and the most likely potential reservations derived from the daily aggregated demand estimation.

The papers focused on adaptive routing, dispatching, and supply optimization generally addressed shorter-term forecasting horizons, predicting demand on an hourly or even 15 min basis. Study [23] uniquely used Wi-Fi data from a university campus in Denmark. Wi-Fi data from six campus buildings were used to generate OD movements, representing aggregated movement counts from one building to another on an hourly basis. This historical hourly OD movement information was then modelled using lagged time-of-day and day-of-week values to produce quantile range predictions for the ranges {5%, 25%, 50%, 75%, 95%}. This study is distinctive both for its use of Wi-Fi aggregated movement data as a proxy for demand and for providing quantile range predictions that represent a predictive demand distribution, which the authors suggest could be leveraged in future work to support dynamic route optimization. Study [37] used call data from a DRT service pilot that operated for 10 months. The call data included OD pairs based on bus stop location and timestamp information for all calls to use the service during the pilot and were aggregated on an hourly basis. Since many OD bus stop pairs showed no demand, the OD information was aggregated into 96 traffic analysis zones (TAZs) to mitigate data sparsity. Calls within the same time window and with the same OD requirements were also combined into a single demand request. This aggregated hourly estimated demand was then used to predict both hourly demand and whether a TAZ would generate any demand during that hour. These outputs could support real-time operational strategies, including vehicle dispatching and relocation, by forecasting where and when DRT demand is likely to occur. Study [38] used public transport bus smart-card data, which provided tap-on/off records for 18 high-frequency bus routes. The data were aggregated at 15 min intervals to forecast future passenger boardings at 15, 30, and 60 min horizons. These predictions were intended to inform vehicle dispatch schedules and expected service levels at each stop and were ultimately used to develop an OD matrix estimation and route-level passenger volumes. These outputs informed a simulator comparing a baseline fixed-route service with two on-demand scenarios. Finally, study [15] used historical demand data containing the time and OD pairs of actual riders of a shared flexible bus service. The data were aggregated at 15 min intervals, ML was used to predict future demand, and the resulting demand predictions were incorporated into a Multi-Agent Reinforcement Learning (MARL) model for dynamic routing optimization.

Only one study [39] focused on demand prediction to support the optimization of cloud resources for DRT service provision. In this study, the public NYC Yellow Cab trip record data were used to model current and past passenger demand in order to predict future demand. The prediction process was conducted in two phases. First, daily booking demand was predicted for the next period; second, actual usage or effective passenger demand, defined as demand minus late or cancelled demand, was derived. This effective demand was then used to predict the number of virtual machines (VMs) required to support the DRT service.

When addressing demand forecasting, it is common to include additional exogenous data, such as weather information, as modelling inputs. In the reviewed forecasting studies, approximately half of the papers augmented historical demand with additional external data. Study [12] added categorical features defining the type of day, such as weekdays, weekends, and holidays, together with numeric weather-related features, including mean, maximum, and minimum temperatures and the number of hours of rainfall per day. Study [37] included date/type-of-day, weather, and TAZ POI data, such as the number of subway stations within each TAZ, alongside OD demand. Their forecasting neural network was also designed to process these datasets differently, as discussed in more detail in the modelling section. Study [23] added a categorical feature indicating when exams were taking place on campus, which improved model performance. Study [38] augmented past passenger counts per stop with cyclical hour-of-day and day-of-week indicators, as well as lagged rolling averages of past demand.

When examining the use of sensor data in the forecasting group, four of the seven studies reported the use of information directly derived from, or informed by, sensors. The most prominent use of sensor data concerned GPS location information, where sensors installed in vehicle navigation devices or mobile phones helped capture vehicle positions, speeds, and travel times. Two studies used GPS-informed data, including vehicle travel times from the taxi dataset used in ref. [39] and ride-request OD locations in the flexible shared-bus dataset used in ref. [15] used tap-on and tap-off records, which enabled bus occupancy tracking through contactless card readers based on radio-frequency technology. In addition, ref. [38] used tap-on/tap-off smart-card records, which enabled the reconstruction of passenger flows through contactless card readers based on radio-frequency technology. Finally, study [23] leveraged passive Wi-Fi sensing to estimate the number of connected devices moving between on-campus buildings as a proxy for demand. The remaining three studies [12,14,37] used OD-based demand data, but it was unclear whether sensors were involved in generating the location information; therefore, these studies should be interpreted as potentially sensor-derived, but not unequivocally sensor-based. Overall, this pattern suggests that sensor-enabled data are becoming an important input for demand forecasting in DRT research, particularly for capturing vehicle locations, travel times, service demand, and passenger flows.

Building on the previous discussion of core demand datasets, temporal horizons, and the use of additional exogenous variables, it is also important to consider whether the reviewed studies provided evidence on the relative contribution of specific input features to forecasting performance. However, this aspect was rarely addressed explicitly. Only one study provided information on which input variables contributed most strongly to the final prediction [12]. For the Seasonal AutoRegressive Integrated Moving Average with eXogenous regressors (SARIMAX) model, the authors reported the coefficients, standard errors, z-scores, *p*-values, and [0.025/0.975] confidence intervals. Based on this information, reservations 7 days before showed the highest statistical significance, while weekday-related input features, such as isWE, isMWF, and isTuTh, also showed high z-scores and *p*-values equal to 0. As only one study within the forecasting group explicitly reported information on variable importance, this aspect was not included as a separate column in Table 8, but is instead discussed here in the text. This represents a difference from the other two groups analyzed in the following sections, where predictor relevance and interpretability were addressed more extensively across multiple studies.

#### 4.1.3. Models and Validation Strategies

Demand forecasting for DRT can be regarded essentially as a time-series forecasting problem, as service demand changes over time in response to temporal patterns such as time of day, weekday, and season. Traditional models for time-series forecasting can be broadly divided into two high-level categories. The first category, which represents the conventional approach to time-series forecasting, includes parametric models that rely on statistical and linear methods to identify and model recurring temporal patterns within the data. These statistical approaches can produce accurate forecasts with relatively limited data when trends are linear or seasonal. However, they require assumptions about the underlying data structure, can be difficult to tune, and are generally less effective in modelling non-linear or complex relationships. The best-known example in this category is ARIMA.

In recent years, ML has emerged as a widely used data-driven alternative to traditional parametric methods. ML approaches are particularly suited to identifying non-linear and complex relationships within historical data and are effective when applied to large datasets with multiple input variables. These approaches can be further divided into classical ML and DL. Classical ML methodologies include tree-based ensemble models, such as RF and LightGBM, which combine multiple decision trees (DTs) to generate predictions through aggregation, bagging, and boosting techniques. DL models, by contrast, are based on neural network architectures and include methods such as Multilayer Feed-Forward Neural Network (MLFF), LSTM, and Convolutional Neural Networks (CNNs).

In the subgroup supporting advanced operational planning and capacity management, study [12] compared four different 7-day-ahead daily demand forecasting models with a baseline naïve model, which calculated daily demand as the weekday average demand from historical data. The models included a SARIMAX model with a rolling forecast. SARIMAX models extend the traditional ARIMAX architecture by adding the ability to capture seasonal trends and patterns. The other models evaluated were an RF Regressor, an LSTM with exogenous variables and a rolling forecast, and a CNN with independent variables. The models were compared using Mean Absolute Error (MAE), Root Mean Square Error (RMSE), Mean Absolute Scaled Error (MASE), and Mean Absolute Percentage Error (MAPE), and the dataset was divided into training, validation, and test sets to ensure comparability of the results. The results showed that the SARIMAX model performed well across all metrics and achieved the lowest RMSE, indicating that it was the most robust in limiting large errors. The LSTM model obtained the best MAE and MASE, while the RF achieved the lowest MAPE. Based on these results, the authors concluded that, for this specific dataset, the temporal patterns were sufficiently linear for the SARIMAX model to perform well, suggesting that the use of more sophisticated DL models was not necessarily justified. The second study in this subgroup [14] compared an LSTM neural network, which included a custom trainable Threshold Layer based on the sigmoid function added as the output of the LSTM, with a basic Moving Average (MA) model. The dataset was split into training and test sets, with the test set consisting of the final month of data. The daily demand predictions were based on the previous four weeks of historical aggregated demand as input. The models were designed to predict the probability that a travel request occurring on a given day belonged to an at-least-once-a-month aggregated travel request corresponding to an identified OD pair. Since the output of the prediction model was fed into a look-ahead offline optimization process, the authors did not rely only on the common Mean Squared Error (MSE) metric but also evaluated the results using Proportion of Correct Predictions (PCPs) and Weight of Correct Predictions (WCPs). It is notable that the study used the outputs of the LSTM model in the offline optimization process and final simulation, even though the LSTM produced slightly worse MSE and PCP values. This choice was motivated by its slightly higher WCP and by the use of a WCP cut-off point, where the LSTM-based model’s best-ranked travel requests were more often correctly predicted than those of the MA model.

In the subgroup concerning adaptive routing, dispatching, and supply optimization, the first paper [23] is distinctive because it evaluated several types of quantile regression (QR) models for demand prediction under uncertainty. QR models offer the advantage of representing prediction uncertainty by providing prediction intervals rather than only single-point estimates. The objective of the study was to predict hourly demand for a DRT shuttle service on a college campus in Denmark, using OD movement information between six campus buildings generated from the campus Wi-Fi system. The authors established the naïve Historical Percentile, based on day-of-week and time-of-day, as the baseline model and compared it with variations of Linear Quantile Regression (LQR), Deep Neural Network (DNN) QR, and GB models. Five LQR models were tested by accounting for seasonality, quantile crossing, exam periods, and by running the model using all ODs together or separately. The DNN and GB models were evaluated using common parameters for all ODs together, as well as separate parameters for each OD pair and for each OD pair plus quantile range. Once again, the models were tested using inputs from all ODs combined and separately. A time-aware train/test split was adopted, and the models were evaluated using three main metrics: Mean Tilted Loss (MTL), the percentage of measured values falling within the 5–95% quantile range, and the mean length of the 5–95% quantile range. Based on these metrics, the multivariate GB model with common parameters for all quantile ranges achieved the best performance. The next study [37] aimed to predict hourly movements between OD pairs representing 96 zones in the city of Yeongjongdo, South Korea, based on historical data from the DRT service I-MOD. The study evaluated nine different models: ARIMA, Lasso regression, Ridge regression, RF, LightGBM, a fully connected neural network (FCN), a CNN, a single-output Convolutional Long Short-Term Memory (ConvLSTM), and a multi-output ConvLSTM. The authors used a time-aware split of the data into training, validation, and test sets, and evaluated the models using F1-score, MSE, and MAE. The multi-output ConvLSTM model achieved the best results across the F1-score and MAE. This model improved demand forecasting performance by estimating demand generation probability, thereby reducing the data sparsity problem, since many OD pairs were expected to have zero demand. The proposed architecture processed different input data types through separate structures: OD data were processed through a CNN-LSTM, weather features through an LSTM, and POI and date features through an FCN. The final outputs were then concatenated, passed through an FCN, and reshaped to provide a probability distribution for each OD pair. The third study in this subgroup [15] incorporated demand forecasts generated by an LSTM neural network at 15 min intervals into a MARL framework. The study used MSE, MAE, and Failed Prediction Ratio (FPR) as evaluation measures and split the input data into three weeks for training and one week for testing. However, the results produced by the LSTM model were not compared with alternative forecasting models.

In the final adaptive routing, dispatching, and supply optimization paper [38] demand prediction results within a hybrid simulation framework were used to support dynamic dispatch, routing, and fleet utilization for on-demand bus services. For the demand prediction task, the results of a Bidirectional Long Short-Term Memory (BiLSTM) neural network were compared with the statistical models ARIMA and Prophet, as well as with three additional neural network models: a unidirectional LSTM, an ANN, and a Gated Recurrent Unit (GRU). The data were split into training (70%), validation (15%), and test (15%) sets, and the models were evaluated using MAPE, coefficient of determination (R^2^), and RMSE. According to the authors, the BiLSTM outperformed the five baseline models across all three metrics and across the three prediction horizons of 15 min, 30 min, and 1 h. The model was also effective in capturing morning and evening peak demand variations.

In the final paper in the demand forecasting group, which focused on cloud resource optimization [39], an MLFF Neural Network was used to predict passenger demand through a two-phase process. First, next-month booking demand was predicted. This information was then used as input, together with cancelled or delayed demand data, to generate a final prediction of actual or effective demand. In their experiments, the authors varied the training/test split between 70% and 90% and tested two MLFF topologies: one with two hidden layers and another with four hidden layers. The MLFF with two hidden layers performed best for both phase I, booking demand prediction, and phase II, expected demand prediction. The study used MSE as the only evaluation metric and did not compare the MLFF model with any alternative models.

#### 4.1.4. Main Findings

The studies focusing on demand forecasting provide examples of how ML can leverage historical demand information, often combined with additional exogenous data, to estimate expected future demand. The most frequently represented applications linked demand forecasting to adaptive routing, dispatching, and supply optimization, advanced operational planning and capacity management, whereas a single study explored its use for cloud-resource management. This latter application area of cloud-resource management, should therefore be considered less established than the other applications identified in this review.

Across the reviewed studies, ML was applied to forecast different forms of future passenger demand at varying temporal resolutions, including daily reservation volumes, hourly or sub-hourly OD demand, probabilistic demand distributions, and effective passenger demand after accounting for cancellations [12,14,15,23,37,38,39].

A common theme emerging from these studies is that both the modelling approach and the temporal horizon are strongly influenced by the intended operational use of the predicted demand. Studies focused on advanced operational planning and capacity management relied on medium-term daily predictions, where temporal patterns were sufficiently stable for traditional statistical models, such as SARIMAX, to compete with more complex DL models [12,14]. By contrast, studies addressing adaptive routing, dispatching, and supply optimization required hourly or 15 min short-term predictions. In these contexts, ML and DL models were generally more suitable because of the non-linear and spatially disaggregated nature of demand [15,23,37,38]. The reviewed studies also highlight that data availability and structure represent significant practical challenges for DRT demand forecasting. Only one study used a publicly available dataset, while the remaining studies relied on non-public operational data from DRT providers, public transit systems, or campus infrastructure [39]. Data sparsity, arising from the large number of OD pairs generating zero demand, was a recurring modelling challenge. This issue was addressed through zone aggregation, probability-based output formulations, and multi-output architectures designed to jointly estimate demand occurrence and volume [23,37]. Half of the forecasting studies also augmented historical demand with exogenous variables, including weather, type-of-day indicators, points of interest, and event calendars, reflecting the well-established sensitivity of transit demand to contextual factors [12,23,37,38].

The reviewed studies further suggest that the value of ML-based demand forecasting in DRT should not be assessed solely through predictive accuracy metrics. In several cases, the forecasting model was embedded directly into a downstream decision-making process, such as offline route optimization, dynamic dispatching, simulation of on-demand service scenarios, or cloud resource scheduling. In these cases, forecast quality was ultimately evaluated not only through standalone error metrics, but also in relation to its impact on operational outcomes [14,15,38,39]. This is particularly evident in ref. [14], where the LSTM model was selected for use in the optimization pipeline despite a marginally lower MSE than the baseline, because its higher weighted prediction quality produced better downstream routing outcomes.

Therefore, demand forecasting represents an anticipatory decision-support layer of ML application in DRT, enabling proactive rather than reactive allocation of vehicles, drivers, and system resources. In this context, forecast quality becomes meaningful when it translates into improved service configuration, scheduling efficiency, or resource utilization.

### 4.2. Operational Predictive Analytics Studies

This section summarizes the studies focused on operational predictive analytics. In DRT systems, service efficiency strongly depends on the effective management of daily operations. Unlike fixed-route public transport, DRT services require continuous decisions regarding stop monitoring, passenger assignment, vehicle dispatching, route adaptation, scheduling, and fleet allocation. Operational complexity further increases when services operate without fixed stops, when users may cancel reservations, cancel late, or fail to appear, and when operators must estimate trip duration, waiting time, level of service (LOS), and the feasibility of matching passengers according to location and time constraints. In this context, ML approaches can support the management and optimization of DRT operations by transforming heterogeneous operational data into actionable predictions.

The operational predictive analytics group included eight studies in which ML approaches were used to support the monitoring, management, or optimization of DRT services. The predicted outcomes included stop type and point of interest, cancellations and no-shows, travel time, waiting time, LOS, request assignment or rejection, and spatiotemporal demand patterns used for service configuration and request-management decisions. Unlike the forecasting studies described in the previous section, these papers did not focus only on predicting future demand volumes. Rather, prediction was used as an operational input to support real-time or near-real-time decisions, improve service reliability, and optimize the interaction between users, vehicles, routes, and schedules.

Table 10 and Table 11 summarize the key information used to analyze this group of studies.

#### 4.2.1. Operational Function

Based on their main operational function, the reviewed studies addressed three main functions: prediction of service reliability and service quality outcomes (three studies), operational monitoring and event interpretation (one study), and prediction embedded in routing, scheduling, and request-management decisions (four studies).

The first function concerned the prediction of service reliability and service quality outcomes. Studies [13,25,42] used predictive analytics to estimate operational outcomes that directly affect service reliability and passenger experience. Study [13] focused on cancellations, late cancellations, and no-shows, addressing the uncertainty generated when booked trips do not become actual trips. Study [25] focused on travel-time prediction in a paratransit service, while study [42] addressed waiting-time and level-of-service prediction for a DRT service for disabled users. Although these studies predicted different outcomes, they share the same operational purpose: reducing uncertainty in service delivery and supporting more reliable decisions regarding vehicle availability, passenger information, and service quality.

The second function concerned operational monitoring and event interpretation, represented by one single study [40]. In this case, the predictive task was used to interpret the behavior of a flexible-stop paratransit service by identifying stop types and nearby points of interest. This application may be particularly relevant for services in which vehicles do not rely exclusively on fixed stops, because automatic event interpretation may help operators understand whether vehicle stops are related to passenger activity, traffic conditions, or the surrounding urban environment. However, given that this subgroup is represented by only one context-specific study, these findings should be considered preliminary, and their applicability to other DRT services remains to be established.

The third and largest function involved prediction embedded in routing, scheduling, and request-management decisions. In this subgroup, predictive outputs were not used only as descriptive or evaluative information, but became inputs for operational decision-making. Studies [16,43] used predicted trip, travel, or service times to improve DAR routing and optimization. Study [41] focused on request assignment and rejection, linking prediction to real-time dispatching and fleet-size planning. Study [44] used spatiotemporal demand information to support customized bus station and route configuration. These studies therefore represent a more advanced operational use of predictive analytics, where ML outputs are directly connected to decisions about which requests to serve, how to schedule vehicles, how to design routes, and how to improve service efficiency.

#### 4.2.2. Data

The data sources used in the operational predictive analytics group reflected the specific operational function addressed by each subgroup.

In the subgroup concerning prediction of service reliability and service quality outcomes, the studies used data that described either reservation behavior or real-time service performance. Study [13] used a paratransit reservation dataset from the Metropolitan Bus Authority, including information on bookings, cancellations, late cancellations, no-shows, trip purpose, subscription status, reservation timing, user characteristics, and previous passenger behavior. These data were suitable for predicting whether a scheduled trip would actually occur, thus supporting reliability-oriented operational decisions. Study [25], instead, used mobile app-collected GPS and stop-related data from onboard paratransit vehicles, combining historical and real-time information such as route length, dwell time, signal delay, traffic conditions, direction, and preceding-vehicle travel time. Study [42] used operational call-taxi log data from a DRT service for disabled users, including the number of calls, number of vacant vehicles, and indicators of spatial imbalance between demand and supply.

In the field of operational monitoring and event interpretation, a single study [40] relied mainly on GPS-based operational traces from Angkot paratransit vehicles. The dataset included latitude, longitude, vehicle identifier, device time, and zero-speed records, which were used to identify and classify stop events. These data were enriched through manual labelling based on Google Maps and Street View, allowing the authors to associate vehicle stops with possible stop types and nearby points of interest.

The subgroup on prediction embedded in routing, scheduling, and request-management decisions used more heterogeneous and decision-oriented datasets. Study [16] used OD trip information and spatial features derived from OpenStreetMap/OSRM, together with traffic-related information from urban sensors. Study [43] relied on GPS and operational data from DAR vehicles, including vehicle locations, arrivals at pick-up and drop-off points, waiting times, loading and unloading times, service times, and travel times. Study [41] used shared e-bike OD data as proxy demand to simulate campus DRT requests. Finally, study [44] used commuter travel-demand data, including origin and destination coordinates and departure and arrival times.

Across the three subgroups, the data used in operational predictive analytics were therefore strongly linked to real-world service operations. Compared with forecasting studies, which mostly relied on historical demand time series, these papers used richer operational datasets, including GPS traces, reservation records, call logs, mobile app data, vehicle-operation records, simulated or proxy OD demand, spatial road-network information, and traffic-related features. This confirms that operational predictive analytics requires data able to describe not only how much demand is expected, but also how users, vehicles, stops, routes, and service constraints interact during DRT operations.

Compared with the forecasting group, operational predictive analytics studies relied more frequently on non-public real-world service data generated during actual DRT, paratransit, DAR, or flexible transport operations. These included reservation logs, call-taxi records, GPS traces, vehicle-location data, waiting times, travel times, service times, cancellations, no-shows, and dispatch-related information. However, not all studies were based exclusively on direct DRT operational data. Some used proxy or derived datasets to reproduce operational conditions, such as shared e-bike OD data to simulate campus DRT requests, while others integrated open or third-party geospatial and network-based information, including OpenStreetMap/OSRM-derived routes, Google Maps/Street View-based manual labelling, and traffic-related data. Therefore, although this group was more strongly grounded in real service operations than the forecasting group, the evidence still combines direct operational records with proxy, simulated, and externally derived data sources.

From a sensor-data perspective, the operational predictive analytics group showed a heterogeneous use of sensor-derived and sensor-related information. Clear sensor-derived data were used in studies based on GPS trackers, mobile-phone GPS, and vehicle-location traces, particularly for stop detection, travel-time prediction, and DAR service-time estimation [25,40,43]. Other studies used geospatial or network-derived data, sometimes combined with sensor-based traffic information, such as OpenStreetMap/OSRM-derived route features and traffic data from urban sensors [16]. In these cases, sensor-derived data helped reconstruct vehicle movement, traffic conditions, route feasibility, and temporal variability in service execution. By contrast, studies [13,42] relied mainly on reservation records, operational logs, call data, and administrative indicators, which are highly relevant for service management but should not be classified as sensor-derived data. Finally, studies [41,44] used spatially explicit OD mobility data, but the underlying data-generation mechanism was not fully specified; therefore, these studies should be interpreted as potentially sensor-derived, but not unequivocally sensor-based. This distinction suggests that operational predictive analytics in DRT is increasingly supported by sensor-enabled data.

#### 4.2.3. Models and Validation Strategies

In the subgroup concerning prediction of service reliability and service quality outcomes, study [13] compared several supervised learning methods, including logistic regression, RF, GB, XGBoost, clustering-based methods, and an ensemble Ordinary Least Squares regression (OLS) approach for daily demand estimation. GB and XGBoost achieved the best performance for predicting reservation outcomes, while the ensemble model was used for aggregated demand estimation. Importantly, this study adopted a more robust validation approach than many others in the group, using rolling-origin time-series evaluation with sequential retraining and a time-aware train–test logic.

Study [25] used an ANN to predict paratransit travel time, comparing the proposed model with historical-average, regression-based, and ANN models using only historical data. The best-performing model combined historical and real-time data. Validation was based on train, validation, and test splits, and performance was assessed using RMSE and MAPE. Study [42] used an Adaptive Neuro-Fuzzy Inference System (ANFIS) to predict waiting time and LOS in a DRT service for disabled users. The model was compared with RF and evaluated through a train–test split. The metrics included RMSE for waiting-time prediction and classification metrics such as accuracy, precision, recall, F1-score, and Receiver Operating Characteristic–Area Under the Curve (ROC-AUC) for LOS prediction.

In the field of research focused on operational monitoring and event interpretation, ref. [40] was the only existing study that used an MLP to classify stop types and points of interest in a flexible-stop paratransit service. The model was compared with alternative MLP configurations, particularly variants without GPS normalization and/or clustering features. Validation was based on a train–test split, and performance was assessed using classification accuracy.

In the subgroup focused on prediction embedded in routing, scheduling, and request-management decisions, study [16] used XGBoost to predict trip duration for origin–destination pairs in a door-to-door DAR service. The model was compared with linear regression, Support Vector Machine (SVM), and RF. Validation combined a train–test split with 5-fold cross-validation (CV), and performance was assessed using R^2^, MAE, and RMSE. Study [43] used ANNs to predict travel time and service time in a DAR problem for elderly and disabled users. The best-performing architecture was a three-layer ANN with ReLU activation functions and stochastic gradient descent optimizer, compared with two-layer ANN alternatives and different activation functions or optimizers. Validation was based on a train–test split, and model performance was assessed using RMSE. Study [41] addressed request assignment and rejection in a simulated campus DRT service developing a DT model and compared it with RF and GB. Validation was performed using 5-fold CV, and model performance was assessed with accuracy, precision, recall, F1-score, and ROC-AUC. Study [44] adopted a different modelling logic by using ST-DP spatiotemporal clustering to identify demand clusters for customized bus planning. The method was compared with K-means and P-DN pairwise density-based spatial clustering. Unlike the other studies, validation was mainly performed through operational performance indicators rather than conventional predictive metrics. Specifically, the study evaluated the impact of the clustering-based method on attendance rate, mean waiting time and mean running time.

#### 4.2.4. Predictor Relevance and Interpretability

Predictor relevance and interpretability were not reported consistently across the operational predictive analytics studies. Therefore, this section focuses only on studies that explicitly reported feature-selection results, variable-importance information, or interpretable predictors associated with model outputs. This distinction is important because, for several studies, the available information concerns the input data used for modelling rather than the relative contribution of individual predictors.

Among the reviewed studies, study [13] provided relevant information on predictors associated with cancellations, late cancellations, no-shows, and aggregated passenger demand in paratransit operations. Relevant predictors included previous passenger behavior, reservation timing, days in advance, subscription status, trip purpose, weekend travel, and demographic characteristics. These variables show that operational uncertainty in reservation-based DRT is not only related to expected demand volume, but also to the probability that booked trips will actually be completed.

Study [25] identified several predictors associated with travel-time prediction in informal paratransit operations, including route length, dwell time, signal delay, recurrent traffic conditions, travel direction, roundabouts, evening peak, and preceding-vehicle travel time. These variables indicate that travel-time prediction in paratransit is strongly influenced by both route characteristics and dynamic traffic conditions.

Park [42] reported interpretable predictors for waiting-time and level-of-service prediction in a DRT service for disabled users. In particular, the number of calls, the number of vacant vehicles, and spatial imbalance indicators between demand and supply emerged as relevant variables. These findings suggest that service quality in DRT for disabled users depends not only on demand intensity, but also on the spatial mismatch between available vehicles and passenger requests.

Finally, study [41] provided one of the clearest variable-importance results in this group, showing that detour factor and ride time were the most important variables for classifying DRT requests as assigned or rejected. This finding highlights that request feasibility in DRT depends strongly on whether a new request can be inserted into the current vehicle schedule without excessive detours or deterioration of passenger service quality.

#### 4.2.5. Main Findings

The studies included in operational predictive analytics show that ML can transform heterogeneous operational data into actionable information for DRT management; indeed, the main contribution of this group is the use of predictive outputs to reduce uncertainty in service operations. Across the reviewed studies, ML was applied to interpret flexible stopping behavior, anticipate cancellations and no-shows, estimate travel time, waiting time and LOS, support request assignment or rejection, and improve routing or service configuration [13,16,25,40,41,42,43,44].

A common finding is that operational performance in DRT depends on the interaction between user behavior, vehicle availability, spatial demand distribution, and route feasibility. Reservation-based services are affected by the uncertainty of whether booked trips will actually occur, as shown by studies predicting cancellations, late cancellations and no-shows [13]. Real-time or flexible services are instead affected by the ability to match requests with available vehicles without increasing detours, delays, waiting times or service quality deterioration, as shown in studies addressing travel-time prediction, waiting-time and level-of-service prediction, request assignment, and routing optimization [16,25,41,42,43].

The reviewed studies also suggest that the value of ML in operational DRT contexts should not be assessed only through predictive accuracy. In several cases, the relevance of the model emerged from its downstream impact on service reliability, routing efficiency, request management, waiting-time reduction, vehicle utilization, or passenger service quality. This is particularly evident in studies where predicted travel or service times were embedded into DAR optimization models, or where request assignment and rejection were directly linked to dispatching and fleet-management decisions [16,41,43]. Similarly, clustering-based approaches showed that data-driven analysis can support the operational configuration of customized services by estimating spatiotemporal demand concentrations and informing stop or route design [44].

Operational predictive analytics represents a decision-oriented layer of ML application in DRT. Its main contribution is to support operators in understanding what is happening in the system, anticipating operational problems, and deciding how to allocate vehicles, manage requests, and adapt service delivery under uncertainty. Therefore, prediction is valuable because it becomes part of the operational decision-making process.

### 4.3. Planning-Oriented Predictive Analytics Studies

This section summarizes the studies focused on planning-oriented predictive analytics. In DRT systems, service efficiency and sustainability are not determined solely by daily operations, but also by well-informed planning decisions. DRT service analysis differs from real-time operational management, as it concerns medium- and long-term decisions that can shape the system, including its structure, geographic coverage, number of vehicles, stopping locations, operational zones, and the analysis of future mobility scenarios [4]. From this perspective, ML methods can support managers and operators by converting large volumes of historical and spatial data into valuable insights for strategic service planning.

In this review, the group of planning-oriented analytics included studies in which ML was applied to support decision-making related to the design, expansion, and configuration of DRT systems. The expected outcomes included defining service zones, identifying spatial mobility patterns, planning service coverage, analyzing accessibility, segmenting users, and supporting the strategic allocation of resources. Unlike the studies discussed in the previous sections, this body of research did not focus on operational forecasting or real-time trip management. Instead, predictive models were used as tools to support strategic planning and policy development aimed at improving the efficiency, coverage, and sustainability of DRT services.

The variety of studies identified in this review and classified as planning-oriented analytics led to their organization into three main areas: Demand Estimation and Ridership Modelling (three studies), Spatial Planning and Service-Area Design (four studies), and Capacity and Fleet Planning (one study). The first subgroup includes studies focused on demand estimation, usage patterns, and service uptake potential. The second includes studies dedicated to spatial analysis, the delimitation of service areas, accessibility, and the territorial configuration of the DRT system. The third focuses on applications related to capacity sizing and strategic fleet planning. This division enabled the studies to be classified according to the different forms of support that ML approaches can provide for DRT system planning.

Table 12 and Table 13 summarize the key information used to analyze this group of studies.

#### 4.3.1. Data

In studies classified as planning-oriented analytics, an analysis was conducted on the types of datasets used to assist in planning functions, as well as the time horizons considered in the modelling. Unlike research focused on short-term operational forecasting, the work of this group was largely based on spatially aggregated data, socioeconomic data, accessibility information, demand histories, and environmental variables to make strategic decisions about the configuration and expansion of DRT systems.

In general, most studies have used private datasets or hybrid datasets that combine various data sources. Among the main types of data used are operational records from DRT services, census data, land use information, accessibility indicators, demographic data, public transport data, spatial data obtained via Geographic Information System (GIS), as well as travel histories or reservations; on the other hand, ref. [46] stood out for using a public dataset widely used in investigations on urban mobility.

Studies within the Demand Estimation and Ridership Modelling subgroup focused primarily on estimating potential demand and service utilization patterns. Ref. [45] used METROLift service data, paratransit service demand census data, and socioeconomic and demographic data to estimate the number of paratransit trips generated in census regions. Study [49] used operational data for on-demand services and census data to estimate daily demand levels in different urban areas and [24] used historical subscription data of a customized bus service combined with built-environment data to estimate and support the planning of passenger flows for different routes and services.

The studies classified under Spatial Planning and Service-Area Design predominantly used spatial data and information related to urban accessibility. Ref. [47] combined monthly bus passenger data, GIS information, land use, and socioeconomic indicators to identify areas suitable for implementing DRT services. Ref. [48] used real public mobility data associated with demographic variables, public transport supply, and accessibility indicators to recommend different types of mobility services for each region analyzed. Simultaneously, ref. [51] utilized the operational demand data of the mybuxi rural service along with certain spatial characteristics to determine pick-up and drop-off patterns, which could help define service areas for DRT in rural contexts. Study [46] also falls within this category and uses historical data of taxi trips to identify travel patterns and support route planning for microtransit and ridesharing services.

Finally, the Capacity and Fleet Planning subgroup, represented by a single study, focused on estimating the operational resources needed for various service contexts. Ref. [50] employed DAR operation simulations in various scenarios, varying factors such as service area, demand density, service level, and operational constraints, in order to estimate the fleet size, vehicle hours, and kilometers driven necessary for strategic operational planning. Its results provide an initial example of the potential contribution of predictive modelling to strategic resource planning.

Regarding time horizon, planning-oriented analytics studies do not primarily focus on short-term forecasts, as is common in operational work. Instead, most analyses use aggregated data on monthly, daily, or long-term historical scales. This data can range from one month to more than two years of temporal coverage, and the models in this group were largely developed to aid key determinations regarding spatial planning, coverage, capacity, configuration of DRT services, among other decision areas.

#### 4.3.2. Predictor Relevance and Interpretability

Studies classified in the planning-oriented analytics group show that the predictions and suggestions for planning made through AI/ML models heavily depend on the spatial, socioeconomic, and physical aspects of the built environment. In addition, some of the studies applied the technique of interpretation to find out the key influencing factors.

The aspects most emphasized in these studies include: the number of people living in an area, how they are distributed, their income and age, whether they have easy access to public transport, how the space is used, the characteristics of buildings and streets, how difficult it is to travel from one place to another, the distance between where people start and end their journeys, the presence of interesting places (such as parks or shops), whether public transport covers the area well, and other indicators that can show how the city is organized. In several studies, these variables were combined with historical operational data from DRT to identify usage patterns and support decisions related to coverage planning and the definition of service areas.

In studies within the Demand Estimation and Ridership Modelling subgroup, a strong dependence on socioeconomic variables and urban environment characteristics was observed for modelling potential demand. In ref. [45], demographic and socioeconomic indicators emerged among the most influential predictors for estimating Americans with Disabilities Act (ADA) paratransit trips in different census regions. Similarly, in ref. [24] they identified that variables related to travel impediments, urban density, and the built environment have a significant influence on demand among potential origin–destination pairs in customized bus services. Ref. [49] used spatial variables and historical mobility patterns to classify demand levels in urban areas.

In the Spatial Planning and Service-Area Design subgroup, spatial factors assumed an even more central role. Ref. [47] was able to analyze the optimal locations to introduce on-demand transport services using different types of land use, scale and commuting demand data, and urban attributes, whereas ref. [48] suggested different types of mobility solutions based on the area’s demographic elements and the region’s public transport supply and accessibility. Meanwhile, ref. [51] showed that spatial characteristics and how people move, as well as pick-up and drop-off patterns, can be used to better understand how the demand for on-demand transportation services works in rural areas and help create more efficient operating areas.

Another relevant aspect identified in the studies was the increasing use of interpretability techniques to understand the individual contribution of predictor variables. Papers such as refs. [48,49] used approaches like SHapley Additive exPlanations (SHAP) values and Partial Dependence Plots (PDPs), making it possible to analyze the impact of factors associated with the spatial, socioeconomic, and built-environment domains on the models’ output. Such approaches helped not only make models more transparent and explainable but also provided valuable insights for urban planning and policymaking.

Overall, from an analysis of the literature presented, it is evident the importance of predictor relevance to planning analytics, showing how demand for DRT, suitability of DRT, and operational factors are highly influenced by spatial, socioeconomic, accessibility, and built-environment factors. Additionally, interpretability helps improve the practical utility of AI/ML models by making their prediction capabilities more applicable to planning activities.

The results show that the demand and adequacy of services depend not only on historical travel patterns, but also on the characteristics of each region, such as territory, society, and structure. Therefore, combining spatial data, urban indicators, and AI/ML techniques becomes essential to help make important decisions about the planning, expansion, and optimization of DRT systems.

#### 4.3.3. Models and Validation Strategies

The studies within the planning-oriented analytics group used a wide range of statistical and ML models to support decision-making related to DRT system planning. Unlike the prediction-oriented category, in which studies mainly focused on forecasting future demand, planning-oriented analytics studies considered broader planning-related objectives. These included demand estimation, the analysis of demand distribution, the identification of critical areas, service configuration, and the allocation of public resources. To address these objectives, the reviewed studies used methods such as regression, classification, clustering, and ensemble learning. In addition, several studies also aimed to improve the interpretability of the results, in order to support more informed planning decisions.

Broadly, the models can be divided into two main categories. The first includes traditional statistical and parametric methods, such as linear regression, Generalised Linear Models (GLMs), and Support Vector Regression (SVR), which were mainly used for explanatory modelling and strategic decision support. The second includes ML-based methods, which have increasingly been adopted because of their ability to model non-linear interactions and complex patterns in spatial, operational, and socioeconomic data. The main models identified in this group included RF, XGBoost, LightGBM, GB, DNNs, and clustering methods such as Density-Based Spatial Clustering of Applications with Noise (DBSCAN).

Within the Demand Estimation and Ridership Modelling subgroup, the main focus was on modelling demand and service usage patterns. Study [45] adopted a mixed approach, using RF to select the relevant set of independent variables, followed by OLS regression to estimate demand for paratransit systems. The authors compared classic linear regression with hybrid models based on Classification and Regression Trees (CART) and RF, applying CV and using adjusted R^2^ as the evaluation criterion. The results showed that automatic variable selection improved model performance while reducing redundancy among explanatory variables.

Similarly, ref. [49] used Bootstrap Aggregating (Bagging) to model trip inputs in DRT, while RF was used to model trip distribution. The authors compared RF, Bagging, ANN, and DNN using a train/validation/test split and 10-fold CV. Model performance was evaluated using accuracy measures and a confusion matrix.

In ref. [24], an XGBoost model was applied to predict stop-to-stop demand for personalized bus services, using both historical user subscription data and built-environment factors. The proposed XGBoost approach was compared with multiplicative models, RF, and GB DTs, using training, 5-fold CV, and testing. The evaluation measures included MAE, RMSE, and pseudo R^2^. The results showed that the proposed XGBoost model achieved better predictive performance, with predictors such as travel impedance and built-environment factors playing an important role in explaining demand between OD pairs.

In the Spatial Planning and Service-Area Design subgroup, most studies focused on spatial analysis tasks and the identification of areas suitable for DRT service implementation. Study [46] used a similarity-based iterative DBSCAN procedure to identify clusters of similar trips and then meta-clusters of recurrent spatiotemporal demand patterns from taxi trip data, used as a proxy for shared mobility demand. These meta-clusters were subsequently used as inputs to a DARP-based optimization model for designing potential microtransit lines. The clustering approach was evaluated using a cluster-quality index.

It was proposed in [47] a methodology based on AutoML_Modified, combining DNN and GB, to identify areas where public transportation was deficient and where DRT could be implemented. The authors compared this model with CART, RF, SVM, Multivariate Adaptive Regression Splines (MARS), and AutoML_Default using 10-fold CV. The adopted metrics included RMSE, MAE, and R^2^, with the hybrid AutoML_Modified model showing the best overall results.

Another important study in this subgroup, ref. [48], used LightGBM to examine the urban and spatial factors influencing DRT demand and service suitability. The model was compared with XGBoost and Categorical Boosting (CatBoost) using a train/test split and 10-fold CV. The evaluation metrics included accuracy, precision, recall, F1-score, and ROC-AUC. The authors also incorporated interpretability methods, such as SHAP and PDP, which made it possible to assess the individual contribution of each urban and spatial variable to the model results.

Similarly, ref. [51] used RF to identify spatial factors associated with DRT demand in rural areas. A distinctive methodological feature of this study was the use of cross-area transfer validation, with training conducted in one region and testing in another. The authors evaluated the results using explained variance and variable-importance analysis.

Finally, for the Capacity and Fleet Planning study, ref. [50] examined GLM and SVR models to support the strategic planning of DAR systems. The study used 5-fold CV to evaluate the models using RMSE and relative prediction error. The results indicated that ML models provided a better capacity to capture the complex relationships between operational variables and system capacity requirements.

Regarding validation strategies, a predominant use of CV was observed, particularly 5-fold and 10-fold CV, in addition to conventional training, validation, and test splits. Unlike studies focused on temporal forecasting, planning-oriented analytics studies prioritized metrics related to explanatory power, classification accuracy, and spatial robustness, including R^2^, RMSE, MAE, accuracy, F1-score, and ROC-AUC.

Another important issue emerging from this group was the increasing attention given to model interpretability. The evaluation of interpretable variables and methods, including SHAP and PDP, was applied in studies such as [48] and [51] to clarify the role of spatial, socioeconomic, and built-environment factors in shaping DRT demand and supply.

#### 4.3.4. Main Findings

Studies within the planning-oriented analytics group illustrate the applicability of ML approaches to decision-making concerning the planning, configuration, and expansion of DRT systems. In contrast to studies that used prediction models for operational forecasting, studies in this category mainly applied predictive models to identify mobility patterns, evaluate demand and accessibility, delineate service areas, and support capacity and fleet planning. The applications identified included OD demand modelling, the identification of areas underserved by public transport, the analysis of built-environment factors associated with service utilization, and the estimation of operational resources required under different operating scenarios [24,45,46,47,48,49,50,51].

One of the main findings emerging from the literature is that model performance is strongly related to the spatial and heterogeneous nature of the data used for DRT planning. In studies focused on Demand Estimation and Ridership Modelling, ensemble learning approaches such as RF, XGBoost, and LightGBM generally provided better results than traditional statistical models, mainly because of their ability to capture non-linear relationships between socioeconomic attributes, travel resistance, and built-environment features [24,48,49]. This suggests that DRT demand depends on multiple interacting spatial and environmental factors.

The reviewed studies also highlight the strong dependence of strategic DRT planning on spatial data and contextual variables. Most studies combined multiple data sources, including operational DRT records, census data, GIS information, built-environment characteristics, land use, accessibility indicators, and public transport data [24,45,47,48,51].

Another recurring aspect was the increasing attention given to model interpretability and explainability. Some studies integrated variable-importance analysis, SHAP values, and other interpretability techniques to understand the effects of urban, socioeconomic, and geographical variables on DRT demand and service suitability [46,48,51]. In the context of urban planning and public transport, these model outputs are particularly relevant because they need to provide practical information for decision-making and policy development.

Moreover, the analysis of the selected literature shows that the use of ML for urban and regional planning is not limited to improving predictive indicators, but also involves applying model outputs to support strategic planning processes. Several studies showed how ML can be used to identify suitable areas, locate stops, extend public transport services, and assess accessibility and capacity [24,47,50]. Therefore, the practical usefulness of these models was mainly evaluated in terms of their ability to generate relevant information to support structural decisions and improve the efficiency and coverage of DRT systems.

Overall, the reviewed studies indicate that planning-oriented analytics represents a strategic layer of ML applications in DRT systems. In this context, models are not used only to predict future demand, but mainly to understand spatial mobility patterns, identify inequalities in access to transport, and support the design of more efficient and accessible services adapted to the territorial and population characteristics of each region.

### 4.4. Gap and Future Directions

The reviewed literature shows that ML-based predictive analytics is increasingly used to support demand forecasting, operational management, and planning-oriented decisions in DRT systems. However, current evidence remains fragmented and only partially transferable to real-world public-transport-oriented DRT services. The main limitations are not related only to model performance, but also to data availability, validation design, generalizability, interpretability, and the limited assessment of operational impact.

A first gap concerns the data used to train and evaluate predictive models. Most studies relied on private, local, or service-specific datasets, including reservation records, call logs, GPS traces, smart-card data, Wi-Fi-derived movements, and operational data that are not publicly available. Only a limited number of studies used public or proxy mobility datasets to infer potential DRT demand, such as taxi records, smart-card transactions, Wi-Fi data, or shared-mobility OD flows [23,38,39,41,46]. While these datasets are useful when direct DRT data are unavailable, they may not fully represent the operational logic of public DRT services. Future research should therefore move towards more transparent and reusable data infrastructures, including benchmark datasets or, at least, standardized reporting of service type, booking rules, spatial units, temporal aggregation, fleet constraints, sensor-derived variables, and preprocessing procedures. In addition, studies should prioritize longer and more temporally diverse observation periods, since short datasets may fail to capture seasonality, holidays, public transport disruptions, exceptional events, and changes in user behavior over time.

A second important limitation concerns validation and generalizability. Many studies were developed within a single case-study context, such as one city, one campus, one rural service, one paratransit agency, or one simulated environment. Moreover, validation strategies were heterogeneous, and some studies relied on conventional train/test splits or CV procedures that may not reflect the temporal and spatial structure of DRT operations. Future studies should adopt validation designs that better approximate real deployment conditions. For demand forecasting, chronological splits, rolling-origin evaluation, and walk-forward validation should become standard. For planning-oriented models, cross-area transfer and external validation across different areas would be particularly valuable. More generally, future research should test whether models trained in one DRT context can be transferred to other cities, service types, fleet sizes, and demand conditions. This would help distinguish between context-specific findings and more generalizable predictive patterns.

A third gap is the limited connection between predictive accuracy and actual service improvement. Most studies evaluated models using standard metrics such as MAE, RMSE, MAPE, MSE, R^2^, accuracy, F1-score, or ROC-AUC. However, good predictive performance does not automatically imply better DRT operations. Some studies embedded predictions into routing, scheduling, fleet-allocation, request-management, or simulation frameworks and evaluated operational outcomes such as travel time, load factor, route efficiency, request assignment, or vehicle utilization [14,15,16,38,41,43,44]. Future work should make this type of evaluation more systematic. In particular, predictive models should be assessed not only through statistical errors, but also through their impact on waiting time, travel time, request acceptance, vehicle occupancy, empty vehicle kilometers, operating costs, service reliability, and accessibility. Moreover, accessibility should be understood not only in terms of service coverage, but also as users’ practical ability to access and use the service. User acceptance, digital inclusion, and the equitable distribution of mobility benefits were rarely examined in the included predictive studies. This is particularly relevant because digital booking systems may create barriers for older adults and users with limited digital access or literacy. Complementary evidence indicates that bridging technologies can facilitate older adults’ use of DRT, while age, gender, and income may influence service adoption and the risk of transport exclusion [54,55]. Future AI-based DRT studies should therefore complement predictive and operational measures with indicators of adoption, usability, digital accessibility, and transport equity.

A fourth direction concerns interpretability and uncertainty. Several studies identified relevant predictors, including previous user behavior, reservation timing, trip purpose, detour factor, ride time, number of calls, vacant vehicles, spatial imbalance indicators, built-environment characteristics, accessibility measures, and socioeconomic variables [13,41,42,46,48,51]. However, predictor relevance was not reported consistently. Since DRT models may influence dispatching, routing, request acceptance, service-area design, and the allocation of public resources, future studies should more systematically include interpretable ML methods, such as feature importance, SHAP values, partial dependence plots, or transparent tree-based models. At the same time, future work should give greater attention to uncertainty-aware prediction. For example, probabilistic forecasting or quantile regression, as explored in ref. [23], may be more useful than point predictions when operators must plan reserve capacity, manage uncertain demand, or evaluate alternative service scenarios.

Finally, future research should move from isolated predictive tasks towards integrated decision-support frameworks. Current studies often address demand forecasting, travel-time prediction, no-show prediction, stop classification, request assignment, service-area design, or fleet planning as separate problems. In real DRT systems, however, these components are interdependent: demand forecasts influence vehicle allocation, travel-time estimates affect routing, cancellation prediction modifies effective demand, and spatial planning shapes future service use. Future work should therefore combine forecasting, operational prediction, optimization, simulation, and policy evaluation within integrated pipelines tested under realistic constraints. In this regard, hybrid metaheuristic approaches developed in other complex logistics optimization contexts may also provide useful methodological insights [56]. This would make ML-based predictive analytics more useful for transport agencies and local authorities, supporting not only accurate prediction but also more efficient, accessible, and publicly oriented DRT services. Furthermore, future deployment-oriented studies should also consider model maintenance and concept drift. DRT demand may evolve after service introduction as users become familiar with the system, as booking rules or service areas change, or as the fixed-route public transport network is modified. Therefore, predictive models should not be treated as static tools, but as adaptive components requiring periodic retraining, drift monitoring, and performance auditing over time.

In summary, future research should not focus only on developing more complex algorithms. The priority should be to make predictive analytics more reliable, interpretable, transferable, and operationally meaningful. Simpler statistical or ML models may remain preferable when they provide comparable performance, greater transparency, and easier implementation. Therefore, model selection should be guided by the specific DRT decision to be supported, the available data infrastructure, the need for interpretability, and the expected public value of the service outcome.

## 5. Conclusions

This systematic review synthesized the current evidence on ML-based predictive analytics for public-transport-oriented DRT systems. The findings show that ML is increasingly being applied to support three main functions: demand forecasting, operational predictive analytics, and planning-oriented decision-making. Overall, the review highlights that predictive analytics in DRT is not limited to estimating future demand, but also supports operational reliability, service configuration, resource allocation, and strategic planning.

With regard to RQ1, the temporal distribution of the included studies indicates that this is a relatively recent and rapidly developing research area. The reviewed papers were published between 2015 and 2025, with most studies appearing from 2020 onwards and a particularly strong concentration between 2023 and 2025. This trend suggests increasing scientific interest in the use of ML and predictive analytics to address the complexity of DRT systems. When considering the thematic distribution over time, the earliest studies were mainly planning-oriented, focusing on demand estimation, service capacity, and strategic configuration. Forecasting studies began to appear later, while operational predictive analytics emerged from 2020 onwards. From 2023 to 2025, the literature expanded across all three categories, with a particularly strong increase in studies addressing forecasting and operational predictive analytics. This suggests a shift from initial planning-oriented applications towards a broader use of ML for both anticipatory demand estimation and operational decision support, although the limited number of early studies prevents firm conclusions about temporal specialization. The novelty of this research field and the recent concentration of publications may reflect both the growing interest of decision-makers in improving public transport and the increasing availability of operational, sensor-derived, and platform-generated mobility data, which provide richer empirical bases for developing and validating ML-based predictive models in DRT contexts.

With regard to RQ2, the publication venues were highly heterogeneous. Most studies were published as journal articles, while a smaller proportion appeared in conference proceedings. The papers were distributed across several transportation-oriented, engineering, AI, and interdisciplinary venues, confirming the multidisciplinary nature of this research field. This dispersion also suggests that ML-based predictive analytics for DRT has not yet consolidated around a limited number of specialized publication outlets.

In response to RQ3, the reviewed studies addressed a heterogeneous set of ML-based predictive analytics tasks, which can be organized into three main functional categories: demand forecasting, operational predictive analytics, and planning-oriented predictive analytics. In the demand forecasting group, ML models were mainly used to anticipate future demand-related quantities over explicit temporal horizons, including daily reservations, hourly or sub-hourly OD demand, passenger volumes, probabilistic demand distributions, and effective demand after cancellations. In the operational predictive analytics group, models addressed outcomes potentially relevant to service execution and real-time or near-real-time decisions, including travel-time and waiting-time estimation, cancellation and no-show prediction, request assignment or rejection, stop and POI classification, level-of-service prediction, and routing or service-configuration decisions. In most cases, however, this operational relevance was assessed retrospectively, offline, or in simulation rather than through prospective deployment in a live service. In the planning-oriented analytics group, predictive models supported medium- and long-term strategic decisions, including ridership estimation, spatial demand modelling, identification of underserved areas, service-area design, accessibility analysis, and capacity or fleet planning. These findings show that ML applications in DRT operate across three interconnected decision levels: an anticipatory level for forecasting future demand, an operational level for managing service reliability and routing constraints, and a strategic level for informing service design, territorial coverage, and resource allocation. A cross-cutting analysis of the service contexts considered in the reviewed studies shows that forecasting and planning-oriented studies covered a broad range of DRT configurations, from paratransit, flexible bus, customized bus, rural DRT, and microtransit services to campus, simulated, and proxy-based on-demand transport scenarios. In these two groups, the type of DRT service mainly defined the application context and the data available for modelling, rather than determining a single dominant predictive task. However, forecasting studies more frequently relied on simulated or proxy-based scenarios, often using non-DRT datasets to reproduce or approximate DRT demand conditions. By contrast, operational predictive analytics studies were more frequently centered on services for elderly, disabled, or reduced-mobility users, suggesting that operational ML applications are particularly relevant in contexts characterized by booking uncertainty, user reliability issues, accessibility constraints, waiting-time sensitivity, and routing or scheduling complexity. Finally, the evidence was unevenly distributed across application subgroups. While some functions were supported by several studies, others, including cloud-resource management, operational event interpretation, and strategic capacity planning, were represented by only one study. Findings from these smaller subgroups should therefore be interpreted as preliminary indications of emerging research directions rather than as consolidated evidence.

With regard to RQ4, the reviewed studies relied on highly heterogeneous data sources and temporal modelling settings, which differed according to the main predictive function addressed. Demand forecasting studies were mainly based on historical demand, reservations, OD movements, smart-card transactions, Wi-Fi-derived mobility patterns, and proxy datasets, and were characterized by explicit prediction horizons ranging from 15 min or hourly forecasts to daily, next-day, or longer-term demand estimates. Operational predictive analytics studies used richer operational and sensor-derived data, including GPS traces, vehicle-location data, call logs, mobile-app data, waiting times, travel times, cancellations, no-shows, service times, and other service-event information. These data were used to model the interaction between users, vehicles, routes, stops, and operational constraints. Planning-oriented studies, by contrast, relied more strongly on spatially aggregated and contextual datasets, including census data, socioeconomic indicators, land-use information, accessibility measures, road-network data, points of interest, GIS layers, and public transport supply variables. In these studies, temporal coverage was less related to short-term forecasting and more often represented the historical basis for strategic decisions on service areas, coverage, capacity, and territorial accessibility. Overall, the evidence indicates that ML-based predictive analytics in DRT depends on the integration of operational, spatial, temporal, contextual, and sensor-derived information. However, most datasets were local, private, and service-specific, while public or proxy datasets were mainly used when direct DRT data were unavailable, limiting reproducibility, cross-study comparability, and transferability across DRT contexts.

In response to RQ5, no single ML technique dominated the field. The reviewed literature included traditional statistical and regression-based models, tree-based and ensemble methods, neural networks and DL architectures, clustering approaches, neuro-fuzzy models, and hybrid frameworks. However, some group-specific tendencies emerged. Forecasting studies were more frequently based on neural and DL architectures, particularly LSTM, ConvLSTM, BiLSTM, and Multilayer Feedforward Artificial Neural Networks (MLFF ANNs), reflecting the temporal and sequential nature of demand prediction tasks. Operational predictive analytics studies showed greater methodological heterogeneity, with approximately half of the selected models based on neural or neuro-fuzzy approaches and several studies relying on tree-based methods such as GB, XGBoost, and DTs. Planning-oriented studies were more frequently associated with tree-based and ensemble models, including RF, XGBoost, LightGBM, and Bagging, as these methods are well suited to modelling non-linear relationships in spatial, socioeconomic, and built-environment data. At the same time, simpler statistical or interpretable models remained relevant, especially when they provided comparable performance, easier implementation, or clearer decision-making insights. Overall, model choice appeared to be strongly task- and context-dependent rather than driven by a single dominant algorithmic family.

With regard to RQ6, model reliability was evaluated using a wide range of validation strategies and performance metrics, reflecting the heterogeneity of the predictive tasks addressed. The most common approaches were train/test splits, train/validation/test splits, and k-fold CV. Forecasting studies more frequently adopted time-aware validation strategies, such as chronological splits, rolling-origin evaluation, or sequential retraining, which are particularly appropriate for temporally ordered demand prediction tasks. Operational predictive analytics studies used more heterogeneous validation designs, often combining conventional splits with task-specific performance indicators, especially when predictions were embedded into routing, request-management, or service-quality applications. Planning-oriented studies more frequently relied on k-fold CV, with one study also adopting cross-area transfer validation to assess transferability across areas. Performance metrics varied according to the task and included MAE, RMSE, MAPE, MSE, R^2^, accuracy, precision, recall, F1-score, ROC-AUC, clustering indices, prediction-interval metrics, and operational indicators. Overall, evaluation practices were not fully standardized, and several studies assessed models mainly through predictive accuracy rather than through their impact on real DRT operations, indicating the need for future studies to combine statistical validation with deployment-oriented evaluation metrics that capture service reliability, efficiency, accessibility, and operational usefulness. Indeed, the use of operational records, real-time variables, or downstream optimization and simulation did not necessarily indicate that a model had been implemented in routine DRT operations. Most studies evaluated predictive performance retrospectively or through internally partitioned datasets, while several relied on proxy or simulation-based evidence. Consequently, the reviewed evidence primarily supports technical feasibility and potential decision-support relevance, while evidence of prospective field performance, operational effectiveness, and sustained model use remains limited.

Finally, in response to RQ7, several gaps remain before ML-based predictive analytics can be considered fully ready for real-world DRT deployment. First, future research should prioritize more transparent and reusable datasets, longer and more temporally diverse observation periods, and clearer reporting of data sources, preprocessing procedures, temporal aggregation, and service characteristics. Second, validation strategies should better reflect real deployment conditions, through time-aware validation for forecasting tasks, spatially robust validation for planning-oriented models, and external validation across different services, cities, and operating contexts. Third, predictive models should be evaluated not only in terms of statistical accuracy, but also according to their downstream impact on DRT operations, including waiting time, travel time, request acceptance, fleet utilization, vehicle occupancy, operating costs, accessibility, and service reliability. These evaluations should also consider user acceptance, digital accessibility, and whether mobility benefits and model-related errors are equitably distributed across different population groups. Greater attention should also be given to interpretability, uncertainty-aware prediction, and the integration of predictive models into operational and planning workflows. Finally, future deployment-oriented studies should treat predictive models as adaptive tools, as DRT demand may evolve over time due to user adaptation, service modifications, or changes in the wider public transport network. Therefore, model maintenance, periodic retraining, drift monitoring, and performance auditing should become central components of real-world ML deployment in DRT systems.

The reviewed evidence indicates that ML-based predictive analytics has the potential to inform more adaptive, efficient, and accessible DRT services. However, it remains insufficient to conclude that these models improve outcomes under routine operational conditions, as prospective field deployment was rarely reported, while external validation and post-implementation monitoring were generally absent or insufficiently documented. This limitation is compounded by the predominance of local, private, and service-specific datasets and by the use of proxy or simulation-based data in part of the evidence base. The methodological assessment further indicated that the strength of the available evidence was uneven, with recurring concerns related to data representativeness, validation design, and analytical procedures. Accordingly, studies linking prediction to routing, optimization, or service planning should primarily be interpreted as offline, simulation-based, or conceptual decision-support evidence rather than as confirmed field deployment. The future development of the field should therefore focus not solely on increasingly complex algorithms, but on models that are reliable, interpretable, transferable, and aligned with the operational and policy objectives of public transport. For researchers, this requires rigorous and reproducible evaluation frameworks, robust baseline comparisons, multiple performance metrics, and prospective assessment of operational impact and user outcomes. For transport agencies and decision-makers, the integration of predictive analytics into decision-support systems should be accompanied by field evaluation, continuous performance monitoring, model maintenance, and assessment of transferability across services and geographical contexts.

As future work, updated systematic reviews should build on this baseline synthesis. As the field is likely to expand with the increasing availability of sensor-derived mobility data, digital booking platforms, and real-time public transport information systems, future reviews should update the evidence base and evaluate whether the methodological gaps identified here are progressively addressed.

## Figures and Tables

**Table 1:** Domain-independent RQs.

ID	Question	Rationale
RQ1	What is the temporal distribution of the papers?	It helps assess the development and temporal trends of scientific research on this topic.
RQ2	What are the most common publication venues for the considered topic?	It helps researchers identify the main publication venues in which studies on this topic are published and to which future work may be submitted.

**Table 2:** Domain-dependent RQs.

ID	Question	Rationale
RQ3	Which ML-based predictive analytics tasks have been addressed in DRT systems, and what functions do they support?	It helps researchers, practitioners, and decision-makers to understand how ML-based predictive analytics has been applied in DRT, and which applications they aim to support.
RQ4	What data sources are typically employed to support prediction tasks in DRT?	It helps understand the characteristics of the employed datasets, including their sources, temporal coverage, and the type of information needed to support ML-based prediction tasks.
RQ5	Which ML techniques are most commonly implemented in this field?	It identifies the predictive techniques most frequently adopted by researchers and helps understand whether the field relies mainly on statistical models, classical ML, DL, or hybrid approaches.
RQ6	How is model reliability evaluated in the literature?	It helps understand how researchers assess model performance and reliability, including the validation strategies and evaluation metrics most commonly adopted.
RQ7	What gaps remain for real-world deployment in DRT operations?	It helps researchers, practitioners, and decision-makers understand which methodological and practical aspects still need to be investigated or improved to support real-world implementation of ML-based models in DRT.

**Table 3:** Quality and applicability of prediction model development. Green **↓**, red **↑**, and grey **//** indicate Low, High, and Unclear concerns, respectively.

Study	Data Sources		Predictors		Outcome		Analysis
	Quality	Applicability	Quality	Applicability	Quality	Applicability	Quality
Chandakas et al. 2020 [12]	**↓**	**↓**	**↓**	**↓**	**↓**	**↓**	**↓**
Zigrand et al. 2023 [14]	**↓**	**↓**	**↓**	**↓**	**↓**	**↓**	**↑**
Peled et al. 2019 [23]	**↑**	**↑**	**↓**	**↓**	**↑**	**↑**	**↓**
Lee et al. 2024 [37]	**↓**	**↓**	**↓**	**↓**	**↓**	**↓**	**↓**
Wu et al. 2024 [15]	**↑**	**↓**	**↓**	**↓**	**↓**	**↓**	**↑**
Liyanage et al. 2025 [38]	**↑**	**↑**	**//**	**↓**	**↓**	**↑**	**//**
Khair et al. 2024 [39]	**↑**	**↑**	**↓**	**↓**	**↑**	**↑**	**↑**
Putri et al. 2020 [40]	**↓**	**↓**	**↑**	**↓**	**↑**	**↓**	**↑**
Acosta Pérez et al. 2020 [13]	**↓**	**↓**	**↓**	**↓**	**↓**	**↓**	**↓**
Ukam et al. 2024 [25]	**↑**	**↓**	**↓**	**↓**	**↓**	**↓**	**↑**
Portell et al. 2025 [16]	**//**	**↓**	**↓**	**↓**	**↓**	**↓**	**↓**
Tandan et al. 2025 [41]	**↑**	**↑**	**↓**	**↓**	**↑**	**↓**	**↑**
Park et al. 2024 [42]	**↓**	**↓**	**//**	**↓**	**↓**	**↓**	**↑**
Belhaiza et al. 2023 [43]	**↓**	**↓**	**↓**	**↓**	**↓**	**↓**	**↑**
Li et al. 2024 [44]	**↑**	**↓**	**↓**	**↓**	**↓**	**↓**	**↓**
Shen et al. 2015 [45]	**↑**	**↓**	**↓**	**↓**	**↓**	**↓**	**↑**
Veve et al. 2022 [46]	**↑**	**↑**	**↓**	**↓**	**↑**	**↑**	**↑**
Oh et al. 2023 [47]	**↑**	**↑**	**↓**	**↓**	**↓**	**↑**	**↑**
Kim et al. 2025 [48]	**//**	**↓**	**↓**	**↓**	**↑**	**↓**	**//**
Alsaleh et al. 2021 [49]	**↓**	**↓**	**↓**	**↓**	**↓**	**↓**	**↓**
Marković et al. 2016 [50]	**↑**	**↓**	**↓**	**↓**	**↑**	**↓**	**↓**
Imhof et al. 2023 [51]	**↓**	**↓**	**↓**	**↓**	**//**	**↓**	**//**
Liu et al. 2023 [24]	**↓**	**↓**	**↓**	**↓**	**↓**	**↓**	**↓**

**Table 4:** RoB and applicability of prediction model evaluation. Green **↓**, red **↑**, and grey **//** indicate Low, High, and Unclear concerns, respectively.

Study	Data Sources		Predictors		Outcome		Analysis
	RoB	Applicability	RoB	Applicability	RoB	Applicability	RoB
Chandakas et al. 2020 [12]	**↓**	**↓**	**↓**	**↓**	**↓**	**↓**	**↓**
Zigrand et al. 2023 [14]	**↓**	**↓**	**//**	**↓**	**↓**	**↓**	**↑**
Peled et al. 2019 [23]	**↓**	**↑**	**↓**	**↓**	**↓**	**↑**	**↓**
Lee et al. 2024 [37]	**↓**	**↓**	**↓**	**↓**	**↓**	**↓**	**↓**
Wu et al. 2024 [15]	**↓**	**↓**	**↓**	**↓**	**↓**	**↓**	**↑**
Liyanage et al. 2025 [38]	**↑**	**↑**	**//**	**↓**	**↓**	**↑**	**↑**
Khair et al. 2024 [39]	**↑**	**↑**	**↑**	**↓**	**↓**	**↑**	**↑**
Putri et al. 2020 [40]	**↑**	**↓**	**↑**	**↓**	**↑**	**↓**	**↑**
Acosta Pérez et al. 2020 [13]	**↓**	**↓**	**↓**	**↓**	**↓**	**↓**	**↓**
Ukam et al. 2024 [25]	**↑**	**↓**	**↑**	**↓**	**↓**	**↓**	**↑**
Portell et al. 2025 [16]	**↓**	**↓**	**↓**	**↓**	**↓**	**↓**	**↓**
Tandan et al. 2025 [41]	**↑**	**↑**	**↑**	**↓**	**↓**	**↓**	**↑**
Park et al. 2024 [42]	**↓**	**↓**	**//**	**↓**	**↓**	**↓**	**↑**
Belhaiza et al. 2023 [43]	**//**	**↓**	**↓**	**↓**	**↓**	**↓**	**↑**
Li et al. 2024 [44]	**↓**	**↓**	**↓**	**↓**	**↓**	**↓**	**//**
Shen et al. 2015 [45]	**↑**	**↓**	**//**	**↓**	**↓**	**↓**	**↑**
Veve et al. 2022 [46]	**↑**	**↑**	**↓**	**↓**	**↑**	**↑**	**↑**
Oh et al. 2023 [47]	**↑**	**↑**	**↑**	**↓**	**↓**	**↑**	**↑**
Kim et al. 2025 [48]	**//**	**↓**	**↓**	**↓**	**↓**	**↓**	**//**
Alsaleh et al. 2021 [49]	**↓**	**↓**	**↓**	**↓**	**↓**	**↓**	**//**
Marković et al. 2016 [50]	**↓**	**↓**	**↓**	**↓**	**↓**	**↓**	**↓**
Imhof et al. 2023 [51]	**↓**	**↓**	**↓**	**↓**	**↓**	**↓**	**//**
Liu et al. 2023 [24]	**↓**	**↓**	**↓**	**↓**	**↓**	**↓**	**↓**

**Table 5:** Cross-tabulation of model family, validation strategy, data availability, temporal coverage, and data provenance by application group.

Dimension and Category	Demand Forecasting, *n* (%)	Operational Predictive Analytics, *n* (%)	Planning-Oriented Predictive Analytics, *n* (%)	Total, *n* (%)
**Main model family**				
Neural networks and deep learning	5 (71.4)	3 (37.5)	0 (0.0)	8 (34.8)
Tree-based and ensemble models	1 (14.3)	3 (37.5)	4 (50)	8 (34.8)
Statistical	1 (14.3)	0 (0.0)	2 (25)	3 (13)
Clustering	0 (0.0)	1 (12.5)	1 (12.5)	2 (8.7)
AutoML	0 (0.0)	0 (0.0)	1 (12.5)	1 (4.3)
Neuro-fuzzy	0 (0.0)	1 (12.5)	0 (0.0)	1 (4.3)
**Validation strategy**				
Time-aware train–validation–test	2 (28.6)	0 (0.0)	0 (0.0)	2 (8.7)
Time-aware train–test/rolling-origin	3 (42.9)	2 (25.0)	0 (0.0)	5 (21.7)
Train–validation–test	1 (14.3)	1 (12.5)	0 (0.0)	2 (8.7)
Train–test	1 (14.3)	3 (37.5)	0 (0.0)	4 (17.4)
Cross-validation only	0 (0.0)	1 (12.5)	3 (37.5)	4 (17.4)
Holdout and cross-validation	0 (0.0)	1 (12.5)	3 (37.5)	4 (17.4)
Spatial transfer	0 (0.0)	0 (0.0)	1 (12.5)	1 (4.3)
No independent out-of-sample validation	0 (0.0)	0 (0.0)	1 (12.5)	1 (4.3)
**Data availability**				
Fully public core modelling data	1 (14.3)	0 (0.0)	1 (12.5)	2 (8.7)
Mixed public and restricted data sources	0 (0.0)	1 (12.5)	2 (25.0)	3 (13.0)
Non-public, restricted, or not explicitly shared	6 (85.7)	7 (87.5)	5 (62.5)	18 (78.3)
**Temporal coverage**				
Short-term: ≤2 months	3 (42.9)	2 (25.0)	3 (37.5)	8 (34.8)
Medium-term: >2–12 months	2 (28.6)	4 (50.0)	1 (12.5)	7 (30.4)
Long-term: >12 months	2 (28.6)	1 (12.5)	1 (12.5)	4 (17.4)
Unclear	0 (0.0)	1 (12.5)	3 (37.5)	4 (17.4)
**Data provenance**				
Direct operational DRT or closely related service data	4 (57.1)	6 (75.0)	5 (62.5)	15 (65.2)
Proxy, indirect or simulation-based data	3 (42.9)	2 (25.0)	3 (37.5)	8 (34.8)

**Table 6:** Cross-tabulation of data availability by data provenance.

Data Availability	Direct Operational or Closely Related Data, *n* (%)	Proxy, Indirect or Simulation-Based Data, *n* (%)	Total, *n*
Fully public core modelling data	0 (0.0)	2 (100)	2
Mixed public and restricted sources	3 (100)	0 (0.0)	3
Non-public, restricted, or not explicitly shared	12 (66.7)	6 (33.3)	18
TOTAL	15 (65.2)	8 (34.8)	23

**Table 7:** Cross-tabulation of temporal coverage by data provenance.

Temporal Coverage	Direct Operational or Closely Related Data, *n* (%)	Proxy, Indirect or Simulation-Based Data, *n* (%)	Total, *n*
Short-term: ≤2 months	3 (20.0)	5 (62.5)	8
Medium-term: >2–12 months	5 (33.3)	2 (25.0)	7
Long-term: >12 months	4 (26.7)	0 (0.0)	4
Unclear or not applicable	3 (20.0)	1 (12.5)	4
TOTAL	15 (100)	8 (100)	23

**Table 8:** Service context, predictive task, and data sources of the demand forecasting studies.

Study	Type of DRT Service	Predictive Task	Data
[12]	Demand-responsive paratransit with prior reservations	Medium-term forecasting of daily reservations/demand	Paratransit reservation platform data and exogenous information (e.g., weather, holidays)
[14]	Suburban dynamic DRT	Next-day forecasting of aggregated travel requests	Historical travel requests, including OD information, and bus stop/service data from a public bus operator
[23]	Campus demand-responsive autonomous mobility	Demand forecasting for OD movements	Campus Wi-Fi OD movement counts
[37]	Station-based DRT	Real-time/short-term forecasting of demand generation probability and demand volume	Historical call/request dataset from the I-MOD DRT service
[15]	Flexible bus service with reservation and immediate requests	OD demand prediction	Historical flexible bus demand data
[38]	Simulated on-demand public transport	Short-term demand forecasting	Public Transport Victoria Smart-card data providing tap-on/off records. Specifically, weekday bus trip records (touch-on/off); OD matrix estimation and route-level passenger volumes
[39]	Simulated cloud-based DRT system	Passenger demand forecasting	Taxi demand datasets from Kaggle, including taxi demand and NYC taxi trip-duration data

**Table 9:** Models, validation strategies, and evaluation metrics of the demand forecasting studies.

Study	Best-Performing Model	Validation	Metrics
[12]	SARIMAX	Time-aware train, validation and test split	MAE 14.03; RMSE 17.85; MASE 0.1034; MAPE 4.24%
[14]	LSTM	Time-aware train and test split	MSE 4.6 × 10^−7^; PCP 71.5%; WCP 27.6%
[23]	GB QR	Time-aware train and test split	Total MTL 433.980; mean ICP_5–95_ 0.883 ± 0.040; mean MIL_5–95_ 34.621 ± 39.883; mean Crossings 0
[37]	Multi-task ConvLSTM	Time-aware train, validation and test split	F1-score 0.9984; MSE 0.0124; MAE 0.0057
[15]	LSTM	Time-aware train and test split	MSE 0.2307; MAE 0.1524; FPR 14.6%
[38]	BiLSTM	Train, validation and test split	MAPE 1.7–4.3% across stops; R^2^ consistently > 0.92; numerical RMSE route-dependent but stable across time periods
[39]	MLFF ANNs	Train and test split	MSE: phase I 1.4316; phase II 2.6183

**Table 10:** Service context, predictive task, and data sources of the operational predictive analytics studies. Where the original study did not explicitly report the information required for a given table field, the corresponding entry is recorded as “Not formally reported”.

Study	Type of DRT Service	Predictive Task	Data	Relevant Predictors
[40]	Paratransit with flexible stopping behavior	Classification of Angkot stop type and POI at stop locations	GPS-based Angkot trip history data, manually labelled using Google Maps/Street View; latitude, longitude, Angkot ID and device time for records with speed = 0 km/h	Not formally reported
[13]	DAR paratransit	Prediction of trip cancellations, late cancellations and no-shows, and aggregated passengers demand	Paratransit reservation dataset from the Metropolitan Bus Authority	Cancellations/no-shows/late-cancellation rates, days in advance, subscription status, trip purpose (work, medical appointment, dialysis, and shopping), weekend travel, age and gender
[25]	Informal paratransit with minibus-taxi	Travel-time prediction	Mobile app-collected GPS and stop-related data from onboard paratransit vehicles on an urban route	Route length, dwell time, signal delay, recurrent traffic conditions, direction, roundabouts, evening peak and preceding-vehicle travel time
[16]	Door-to-door DAR for people with reduced mobility	Trip duration prediction for origin–destination pairs	OD trips from OpenStreetMap/OSRM with spatial features; traffic data from urban sensors	Not formally reported
[41]	Simulated campus DRT	Classification of DRT requests as assigned or rejected under different fleet-size scenarios	Shared e-bike OD data	Detour factor and ride time
[42]	DRT for disabled users	Prediction of waiting time and LOS of DRT for the disabled	Operational DRT call-taxi log data	Number of calls, number of vacant vehicles and spatial imbalance indicators between demand and supply
[43]	DAR for elderly and disabled users	Prediction of travel time and service time for DARP	GPS and operational data from DAR vehicles, including vehicle locations, arrivals at pick-up/drop-off points, waiting times, loading/unloading times, and travel times	Not formally reported
[44]	Demand-responsive customized bus service	Estimation of spatiotemporal demand clusters for customized bus planning	Commuter travel demand data including origin/destination coordinates and departure/arrival times	Not formally reported

**Table 11:** Models, validation strategies, and evaluation metrics of the operational predictive analytics studies.

Study	Best-Performing Model	Validation	Metrics
[40]	MLP	Train and test split	Accuracy 95%
[13]	GB and XGBoost for reservation outcomes; OLS ensemble for daily demand estimation	Rolling-origin time-series evaluation with sequential retraining and time-aware train and test split	Binary classification accuracy XGBoost 78.9%; multiclass GB accuracy 76.0%; OLS demand ensemble MAPE 7.4% and R^2^ 0.98
[25]	ANNs	Train, validation and test split	RMSE 0.92 min; MAPE 14%
[16]	XGBoost	Train and test split with 5-fold CV	R^2^ 0.98; MAE 28.12 s; RMSE 37.51 s
[41]	DT	5-fold CV	Accuracy 0.73–0.83 across the evaluated fleet-size scenarios
[42]	ANFIS	Train and test split	Overall accuracy 90%; mean ROC-AUC 92%; class F1-score: Good 0.93, Moderate 0.85, Bad 0.93; class Precision: Good 0.93, Moderate 0.83, Bad 0.94; class Recall: Good 0.93, Moderate 0.86, Bad 0.91
[43]	ANNs	Train and test split	RMSE 0.31806
[44]	ST-DP spatiotemporal clustering	Time-aware train–test split with operational performance evaluation	Attendance rate 88.4%; mean waiting time 278.93 ± 1.77 s; mean running time 3397 ± 24.07 s

**Table 12:** Service context, predictive task, and data sources of the planning-oriented predictive analytics studies. Where the original study did not explicitly report the information required for a given table field, the corresponding entry is recorded as “Not formally reported”.

Study	Type of DRT Service	Predictive Task	Data	Relevant Predictors
[45]	ADA paratransit	Prediction of ADA paratransit travel demand at census-tract level	METROLift Houston trips and Census data from the factfinder website	Fraction of senior citizens, Average household size, Fraction of African Americans, Fraction of Hispanics, Annual household income, Fraction of males, Median age, Fraction of households with a family member with a disability and Total population
[46]	Microtransit/customized flexible transport	Modelling of recurrent spatiotemporal demand patterns	NYC Taxi and Limousine Commission trip data	Not formally reported
[47]	Potential DRT bus-stop allocation for public-transport vulnerable areas	Prediction of bus passenger demand	Monthly bus passenger data collected by web crawling, bus stop/location data, GIS spatial layers, socioeconomic, public-transport and land-use data	Not formally reported
[48]	Demand-responsive mobility service planning: fixed-route bus, DRT-bus and DRT-taxi service types	Classification of spatial zones into fixed-route bus, DRT-bus or DRT-taxi suitability	Public mobility service usage data combined with 500 m × 500 m grid-cell demographic, vehicle ownership, driver-license, public transport supply and accessibility indicators	Walking distance to the nearest bus stop, Total population, Elderly population, Walking distance to public facilities, Railway station accessibility, Hospital accessibility, Licensed driver proportion and Child population density
[49]	On-demand transit replacing a late-night fixed-route bus	Prediction of daily trip production and distribution levels at dissemination areas	On-demand transit operational data and census data	Land-use type
[50]	DAR for elderly and disabled users	Prediction of required DAR system capacity: fleet size, vehicle-hours and vehicle-kilometres	Simulated DAR planning scenarios generated from service-region, demand, level-of-service and operator-constraint variables; real-world DAR scenarios	Not formally reported
[51]	Rural DRT with virtual stops	Spatial prediction of DRT pick-ups and drop-offs at zone level	Passenger demand of mybuxi operator and spatial data	Number of inhabitants, Distance to train station, Presence of restaurants
[24]	Demand-responsive customized bus	Stop-to-stop customized bus ridership modelling	User subscription data from largest customized bus service provider, and environment data	Trip distance, Trip fare, Distance to city center, Distance to nearest metro station, Population density, Land-use mix, Road density, Job accessibility, Amenity accessibility

**Table 13:** Models, validation strategies, and evaluation metrics of the planning-oriented predictive analytics studies.

Study	Best-Performing Model	Validation	Metrics
[45]	OLS	CV	Adjusted R^2^ 0.420
[46]	DBSCAN	Not explicitly reported	Custom internal cluster-quality index; no standard quantitative clustering performance metric was reported
[47]	AutoML_Modified, combining DNN and GBM	10-fold CV	RMSE 0.146; MAE 0.085; R^2^ 0.700
[48]	LightGBM	Train and test split with 10-fold CV	Accuracy 0.9189; precision 0.9284; recall 0.9690; F1-score 0.9482
[49]	Bagging for trip production; RF for trip distribution	Train, validation (10-fold CV) and test split	Accuracy; confusion matrix Trip production: Bagging overall accuracy 64%; Trip distribution: RF overall accuracy 72%.
[50]	GLM	5-fold CV	RMSE: 2.25 vehicles, 28.75 vehicle-hours, 2294 vehicle-km; mean relative error: 4.03%, 5.94%, 13.45%
[51]	RF	Within-area train/test + cross-area transfer (train area A → test area B)	Variable importance; corrected overall predictive-performance metrics not reported
[24]	XGBoost	Train, validation (5-fold CV) and test split	Morning: pseudo-R^2^ 0.476; MAE 1.193; RMSE 1.493. Evening: pseudo-R^2^ 0.478; MAE 1.206; RMSE 1.482

## Data Availability

No new primary data were generated in this study. All data extracted from the included studies and supporting the findings of this systematic review are reported within the article and its tables.

## Supplementary Materials

The following supporting information can be downloaded at: https://www.mdpi.com/article/10.3390/s26154945/s1, Table S1: PRISMA 2020 Checklist.

## Abbreviations

The following abbreviations are used in this manuscript:

ADA	Americans with Disabilities Act
AI	Artificial Intelligence
ANFIS	Adaptive Neuro-Fuzzy Inference System
ANN	Artificial Neural Network
ARIMA	AutoRegressive Integrated Moving Average
AutoML	Automated Machine Learning
Bagging	Bootstrap Aggregating
BiLSTM	Bidirectional Long Short-Term Memory
CART	Classification and Regression Tree
CatBoost	Categorical Boosting
CNN	Convolutional Neural Network
ConvLSTM	Convolutional Long Short-Term Memory
CV	Cross-validation
DAR	Dial-a-Ride
DARP	Dial-a-Ride Problem
DBSCAN	Density-Based Spatial Clustering of Applications with Noise
DL	Deep Learning
DNN	Deep Neural Network
DRT	Demand-Responsive Transport
DT	Decision Tree
ETA	Estimated Time of Arrival
FCN	Fully Convolutional Network
FPR	Failed Prediction Ratio
GB	Gradient Boosting
GBM	Gradient Boosting Machine
GIS	Geographic Information System
GLM	Generalised Linear Model
GPS	Global Positioning System
GRU	Gated Recurrent Unit
ICP_5–95_	Interval Coverage Probability for the 5–95% quantile interval
ITS	Intelligent Transportation Systems
LightGBM	Light Gradient Boosting Machine
LOS	Level of Service
LQR	Linear Quantile Regression
LSTM	Long Short-Term Memory
MA	Moving Average
MAE	Mean Absolute Error
MAPE	Mean Absolute Percentage Error
MARL	Multi-Agent Reinforcement Learning
MASE	Mean Absolute Scaled Error
MARS	Multivariate Adaptive Regression Splines
MIL_5–95_	Mean Interval Length for the 5–95% quantile interval
ML	Machine Learning
MLFF	Multilayer Feed-Forward Neural Network
MLP	Multilayer Perceptron
MSE	Mean Squared Error
MTL	Mean Tilted Loss
NYC	New York City
OD	Origin–destination
OLS	Ordinary Least Squares
PCP	Proportion of Correct Predictions
PDP	Partial Dependence Plot
POI	Point of Interest
PRISMA	Preferred Reporting Items for Systematic Reviews and Meta-Analyses
QR	Quantile Regression
R^2^	Coefficient of determination
RF	Random Forest
RMSE	Root Mean Square Error
RoB	Risk of Bias
ROC-AUC	Receiver Operating Characteristic–Area Under the Curve
RQ	Research Question
SARIMAX	Seasonal AutoRegressive Integrated Moving Average with eXogenous regressors
SHAP	SHapley Additive exPlanations
ST-DP	Spatiotemporal clustering based on DBSCAN and PAM
SVM	Support Vector Machine
SVR	Support Vector Regression
TAZ	Traffic Analysis Zone
VM	Virtual Machine
WCP	Weight of Correct Predictions
XGBoost	eXtreme Gradient Boosting
